# High sensitivity-low cost detection of SARS-CoV-2 by two steps end point RT-PCR with agarose gel electrophoresis visualization

**DOI:** 10.1038/s41598-021-00900-8

**Published:** 2021-11-04

**Authors:** Solange Figueroa, Byron Freire-Paspuel, Patricio Vega-Mariño, Alberto Velez, Marilyn Cruz, Washington B. Cardenas, Miguel Angel Garcia-Bereguiain

**Affiliations:** 1 One Health Research Group, Universidad de Las Americas, Quito, Ecuador; 2 Agencia de Regulación y Control de la Bioseguridad y Cuarentena para Galápagos, Puerto Ayora, Ecuador; 3 Laboratorio para Investigaciones Biomédicas, Escuela Superior Politécnica del Litoral, Guayaquil, Ecuador

**Keywords:** Microbiology, Virology, SARS-CoV-2

## Abstract

More than one year since Coronavirus disease 2019 (COVID-19) pandemic outbreak, the gold standard technique for severe acute respiratory syndrome coronavirus 2 (SARS-CoV-2) detection is still the RT-qPCR. This is a limitation to increase testing capacities, particularly at developing countries, as expensive reagents and equipment are required. We developed a two steps end point RT-PCR reaction with SARS-CoV-2 Nucleocapsid (N) gene and Ribonuclease P (RNase P) specific primers where viral amplicons were verified by agarose gel electrophoresis. We carried out a clinical performance and analytical sensitivity evaluation for this two-steps end point RT-PCR method with 242 nasopharyngeal samples using the CDC RT-qPCR protocol as a gold standard technique. With a specificity of 95.8%, a sensitivity of 95.1%, and a limit of detection of 20 viral RNA copies/uL, this two steps end point RT-PCR assay is an affordable and reliable method for SARS-CoV-2 detection. This protocol would allow to extend COVID-19 diagnosis to basic molecular biology laboratories with a potential positive impact in surveillance programs at developing countries.

## Introduction

The Coronaviruses Disease 2019 (COVID-19) pandemic, caused by the infection of Severe Acute Respiratory Syndrome Coronavirus 2 (SARS-CoV-2), has challenged public health systems worldwide since the initial outbreak in the Chinese city of Wuhan in December 2019^1,2^. By September 1st 2021, SARS-CoV-2 has caused more than 218 million infections and 4.5 million deaths worldwide (https://coronavirus.jhu.edu/map.html). Developing countries from Latin America has been deeply affected by COVID-19 pandemic, as many of the public health systems on these countries have been traditionally neglected in terms of government funds. Moreover, there are additional factors of sensitivity to SARS-CoV-2 in Latin America such as HLA haplotypes specific to the region^3^. So, when COVID-19 pandemic landed, testing laboratories and hospitals were quickly overflow^4,5^.

Since the outbreak of COVID-19 pandemic, the gold standard technique for SARS-CoV-2 infection detection is RT-qPCR. Several in vitro diagnosis RT-qPCR kits are available in the market for SARS-CoV-2 detection. For instance, the USA Center for Disease Control and Prevention (CDC) RT-qPCR protocol is based on N1 and N2 gene targets to detect SARS-CoV-2 and RNase P gene target for RNA extraction quality control, and it is considered a gold standard method for high sensitivity SARS-CoV-2 detection^6–10^. Unfortunately, routine diagnosis by RT-qPCR is still a limitation for many laboratories, especially in developing countries like Ecuador, where the cost of a RT-qPCR device is over 30.000 USD (for reference, the minimum monthly wage is 400 USD) and the costs of reagents like primers, probes and RT-qPCR master mix are even higher than in developing countries due to the importation fees. Besides, the current worldwide high demand for reagents supplies and equipment, and also the few supplier companies (mostly located at high income countries), challenge a timely effective detection of SARS-CoV-2 positive cases.

In order to increase the SARS-CoV-2 testing capacities, we herein describe a new method for SARS-CoV-2 detection using a two steps end point RT-PCR protocol, where conventional non real time thermal cycler, the same set or primers for N1, N2 and RNase P targets included on the CDC RT-qPCR protocol, and agarose gel electrophoresis are used. Moreover, we carried out a clinical performance and analytical sensitivity evaluation for this method, using the CDC RT-qPCR protocol as a gold standard technique.

## Material and methods

### Study design

242 clinical specimens (nasopharyngeal swabs collected in 0.5 mL TE pH 8 buffer) were included in this study. Also, 11 negative controls (TE buffer [pH 8.0]) were included as control for carryover contamination, one for each set of RNA extractions. Moreover, a high viral load positive sample for each of the following respiratory viruses were tested to address cross reactivity: Influenza A, Influenza B, Respiratory Syncytial Virus (RSV) and Human Metapneumovirus (HMPV).

### RNA extraction

**Figure 1:**
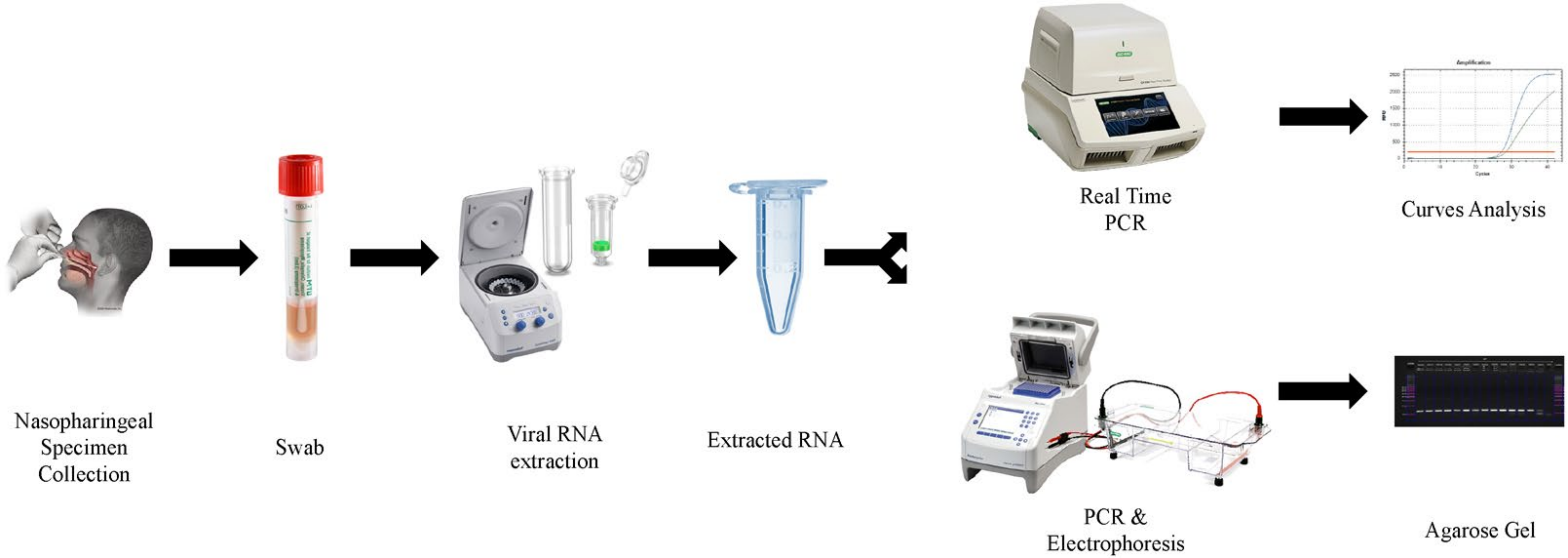
Flow chart for sample processing by CDC RT-qPCR protocol and two step end point RT-PCR for SARS-CoV-2 detection.

All the samples were processed with AccuPrep Viral RNA extraction kit IVD (Bioneer, South Korea), following manufacturer’s instructions. The same RNA aliquot was used for both investigated protocols (RT-qPCR and two step end point RT-PCR). The samples flow chart is presented in Fig. 1.

### SARS-CoV-2 detection using the CDC RT-qPCR protocol

All the samples included in the study were tested following an adapted version of the CDC RT-qPCR protocol: (a) using Accupower Viral RNA extraction kit (Bioneer, South Korea) as an alternative RNA extraction method; (b) using CFX96 BioRad instrument^11–19^; (c) using a N1/N2/RNase P multiplex assay^11^. The criteria for positivity was a Ct ≤ 40 for N1 and N2 targets simultaneously^6,7^; to consider a sample as negative, a Ct ≤ 40 for RNase P target is needed in the absence of N1 and N2 amplification.

### Two-step end point RT-PCR for SARS-CoV-2 detection

Same concentrations for N1, N2 and RNase P primers than for the CDC RT-qPCR protocol were used. We carried out a two step RT-PCR protocol using High Capacity Superscript cDNA Reverse Transcription Kit (Applied Biosystems, USA) for retrotranscription (RT) and DreamTaq Green PCR Master Mix (Thermo Scientific, USA) for PCR. Both the RT and PCR were performed in a Mastercycler Pro (Eppendorf, USA) thermal cycler. Retrotranscription was performed with 5uL of RNA and 5uL of RT master mix using the following conditions: 25 °C 2 min, 37 °C 120 min, 85 °C 5 min. After RT reaction, 4uL of nuclease free water were added to the 10uL of cDNA in order to avoid possible PCR inhibitions. Then, PCR was performed in a final volume of 15ul using 3uL of the diluted cDNA as template. The PCR conditions were: 95 °C 3 min; 38 cycles of 3 step: 95 °C 30 s; 58 °C 30 s; 72 °C 30 s; and final step 72 °C 5 min. Amplicons were visualized in 3% agarose gels stained with SYBR Safe. The band sizes were 72 bp and 67 bp, for N1 and N2, respectively; and 65 bp for RNase P. As it was detailed for the CDC RT-qPCR protocol^7,8^, the criteria for positivity was the presence of both a N1 and N2 targets bands simultaneously in the agarose gels. When only N1 or N2 bands appears, either PCR repetition or new RNA extraction are necessary to confirm results for those inconclusive samples, as recommended by the CDC guidelines for RT-qPCR SARS-CoV-2 detection^7^.

### Analytical sensitivity

Limit of detection (LoD) was performed using the 2019-nCoV N positive control (IDT, USA) provided at 200.000 genome equivalents/uL for the SARS-CoV-2 CDC RT-qPCR protocol. As 40uL of elution buffer and 0.2 mL of sample are used in the RNA extraction protocol, a 200 conversion factor is applied to change LoD units from copies/uL of RNA extraction solution to copies/mL of nasopharyngeal sample.

### Ethics statement

All samples have been submitted for routine patient care and diagnostics. Ethics approval was not sought because the study involves laboratory validation of test methods and the secondary use of anonymous pathological specimens that falls under the category ‘exempted’ by Comité de Etica para Investigación en Seres Humanos" from "Universidad de Las Américas".

## Results

### Clinical performance of the two steps end point RT-PCR method compared to the SARS-CoV-2 CDC RT-qPCR protocol

**Figure 2:**
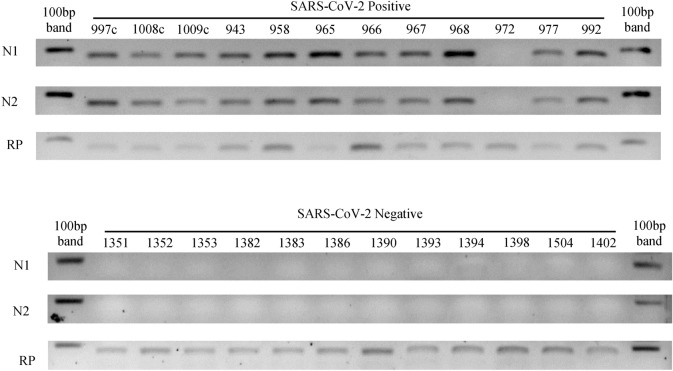
Agarose gel (3%) electrophoresis for SARS-CoV-2 positive and negative samples. N1 and N2 amplicons correspond with viral amplicons for CDC RT-qPCR protocol. RNase P (RP) amplicons show RNA extraction quality control. Numbers indicates samples code (see Supplementary Material 1 for Ct values and viral loads). 20 samples are illustrated out of the total 242 ones (See Supplementary Material 3).

242 samples were tested for SARS-CoV-2 following both protocols described in the methods. For the CDC RT-qPCR protocol, 122 samples were SARS-CoV-2 positive and 120 samples were SARS-CoV-2 negative (Table 1; Supplementary Material 1). 116 out of the 122 positive samples yielded a clear band at the expected size for N1 and N2 in the agarose gel electrophoresis following the two steps end point RT-PCR protocol (Fig. 2; for whole agarose gel pictures see Supplementary Material 3). So, the sensitivity of the two steps end point RT-PCR was 95.1% (116/122). Moreover, bands for N1 and N2 were simultaneously observed at the agarose gel for 5 of the 120 SARS-CoV-2 negative samples (Supplementary Material 2 and 3). So, the specificity of the two steps end point RT-PCR was 95.8% (115/120). Cross reactivity with other respiratory viruses like influenza A, influenza B, RSV and HMPV was excluded as no band at the N1 and N2 amplicons size was observed for previously confirmed positive samples for those viruses (see Supplementary Material 4).

**Table 1:** Clinical performance of two step end point RT-PCR compared to the CDC RT-qPCR protocol for SARS-CoV-2 detection.

Sample type	end point RT-PCR ( +)	end point RT-PCR (-)	Total samples
RT-qPCR ( +)	116 (95.1%)	6	122 SARS-CoV-2 ( +)
RT-qPCR ( −)	5	115 (95.8%)	120 SARS-CoV-2 (-)

Cohen's κ was run to determine the level of agreement between results obtained using two step end point RT-PCR and CDC RT-qPCR protocol for SARS-CoV-2 detection. There was almost perfect agreement between results obtained with both methodologies as indicated by a Cohen's κ = 0.909 (*P* < 0.001).

### Analytical sensitivity for the two step end point RT-PCR method for SARS-CoV-2 detection

**Figure 3:**
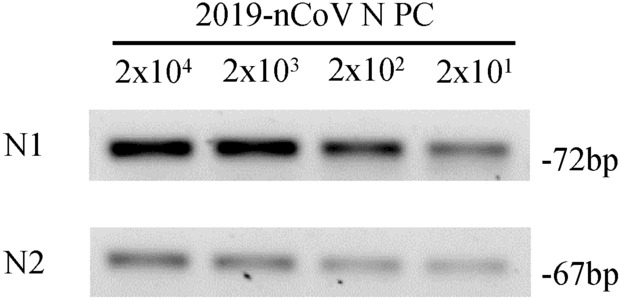
Analytical sensitivity for the two steps end point RT-PCR method for SARS-CoV-2 detection. RT-PCR and agarose gel (3%) electrophoresis for "2019-nCoV N PC" positive control for N1 and N2 amplicons at the detailed concentrations were replicated 5 times. Values correspond to viral loads in copies/uL.

The viral loads detailed in Supplementary Material 1 were calculated running a calibration curve with 2019-nCoV N positive control (IDT, USA). The LoD for the CDC RT-qPCR protocol was set at 1000 viral copies/mL of sample (or 5 viral copies/uL of RNA extraction solution) on previous studies^6,10–17^. As it is detailed in Fig. 3, the LoD for the two step end point RT-PCR is estimated to be 20 viral copies/uL of RNA extraction (4000 viral copies/mL of sample), as the five replicates done at this concentration yielded a clear band on the agarose gel electrophoresis.

## Discussion

Our results support that the detection of SARS-CoV-2 using the two step end point RT-PCR method described here, followed by agarose gel electrophoresis, is a reliable alternative to the gold standard RT-qPCR tecnique. With a specificity of 95.8%, a sensitivity of 95.1% and a LoD of 20 copies/uL, this affordable method has a similar clinical performance to most of the RT-qPCR commercial kits, usually more expensive^10–13,17–21^. Moreover, as it has also been recently reported that only patients with viral loads over 1 million copies/mL (5000 copies/uL of RNA extraction on our experimental conditions) would be infectious, this two step end point RT-PCR method would virtually detect 100% of the infectious SARS-CoV-2 positive cases^22,23^.

Although a few studies have already reported end point RT-PCR methods for SARS-CoV-2 detection^24–27^, the present work was pioneer as it was already published as a preprint at MedRxiv in May 2020^27^. Actually, only one of those reports has already been published at a peer review journal, while the others are still at a preprint stage^24–26^. Moreover, with a sample size of 242, including 122 SARS-CoV-2 positive samples, this study is to our knowledge the most statistically significant, as the previous ones were done with substantially smaller samples size of 43 and 30^24–26^. Also, this study is the only one using the primers set from the CDC-RT-qPCR protocol for SARS-CoV-2 detection and visualizing the results by agarose gel electrophoresis. Nevertheless, we found a sensitivity value for this two step end point RT-PCR method in the same range than those other studies, where sensitivity values of 94.2%, 96.9% and 100% were described^24–27^.

In summary, we herein describe a two step end point RT-PCR method as reliable for SARS-CoV-2 detection as most of the commercial kits for SARS-CoV-2 RT-qPCR. This two step end point RT-PCR protocol for SARS-CoV-2 detection would be helpful to reduce cost associated to SARS-CoV-2 surveillance and to improve diagnosis at locations lacking of Real Time PCR devices but with more basic molecular biology equipment, as it is usually the case for laboratories at middle and low income countries.

## Supplementary Information

Supplementary Information 1.

Supplementary Information 2.

Supplementary Information 3.

Supplementary Information 4.
