# Supplementary material for: High sensitivity-low cost detection of SARS-CoV-2 by two steps end point RT-PCR with agarose gel electrophoresis visualization
Source: Sci Rep. 2021 Nov 4;11:21658. doi: 10.1038/s41598-021-00900-8 (PMC8568942; doi:10.1038/s41598-021-00900-8)
Supplement: Supplementary file 2 — Supplementary Information 2. [file 41598_2021_900_MOESM2_ESM.pdf]

Supplementary material 2. Full agarose gel electrophoresis pictures for all the samples included on the study.

SARS-CoV-2 Positive Samples

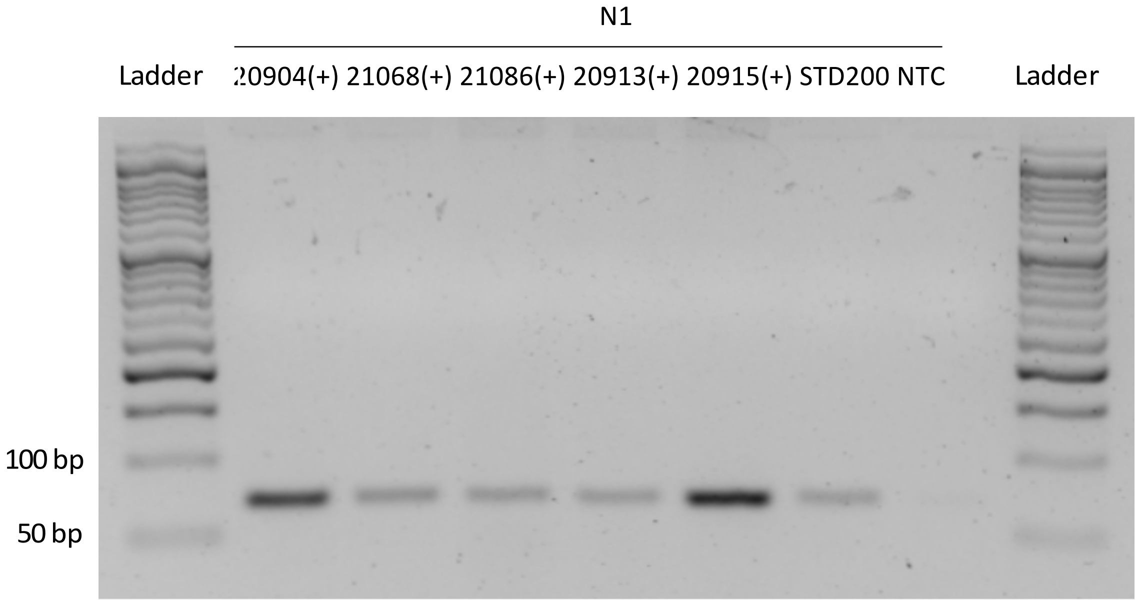

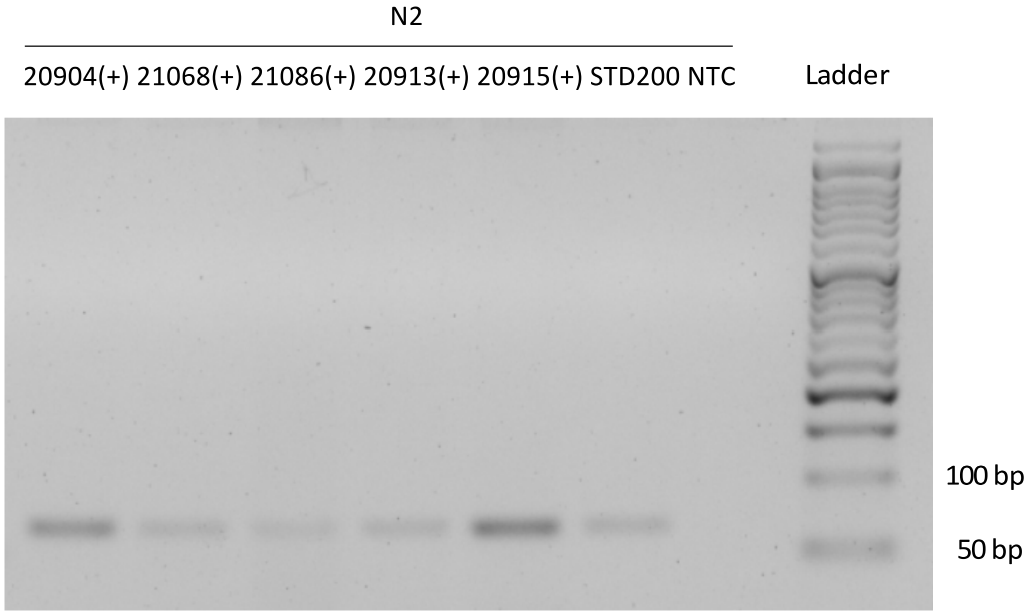

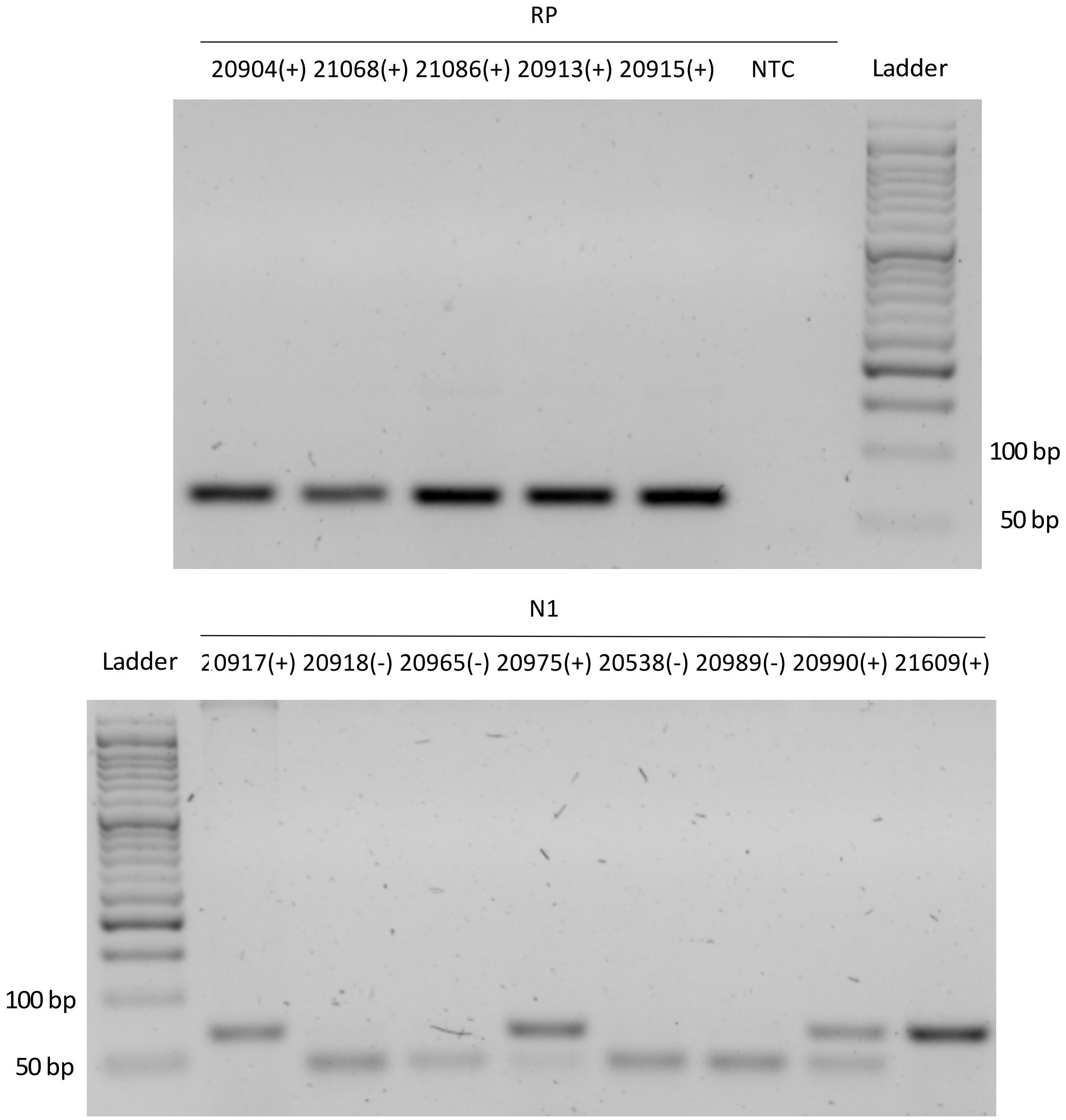
RP
20904(+) 21068(+) 21086(+) 20913(+) 20915(+) NTC
Ladder
100 bp
50 bp
N1
Ladder
20917(+) 20918(-) 20965(-) 20975(+) 20538(-) 20989(-) 20990(+) 21609(+)
100 bp
50 bp

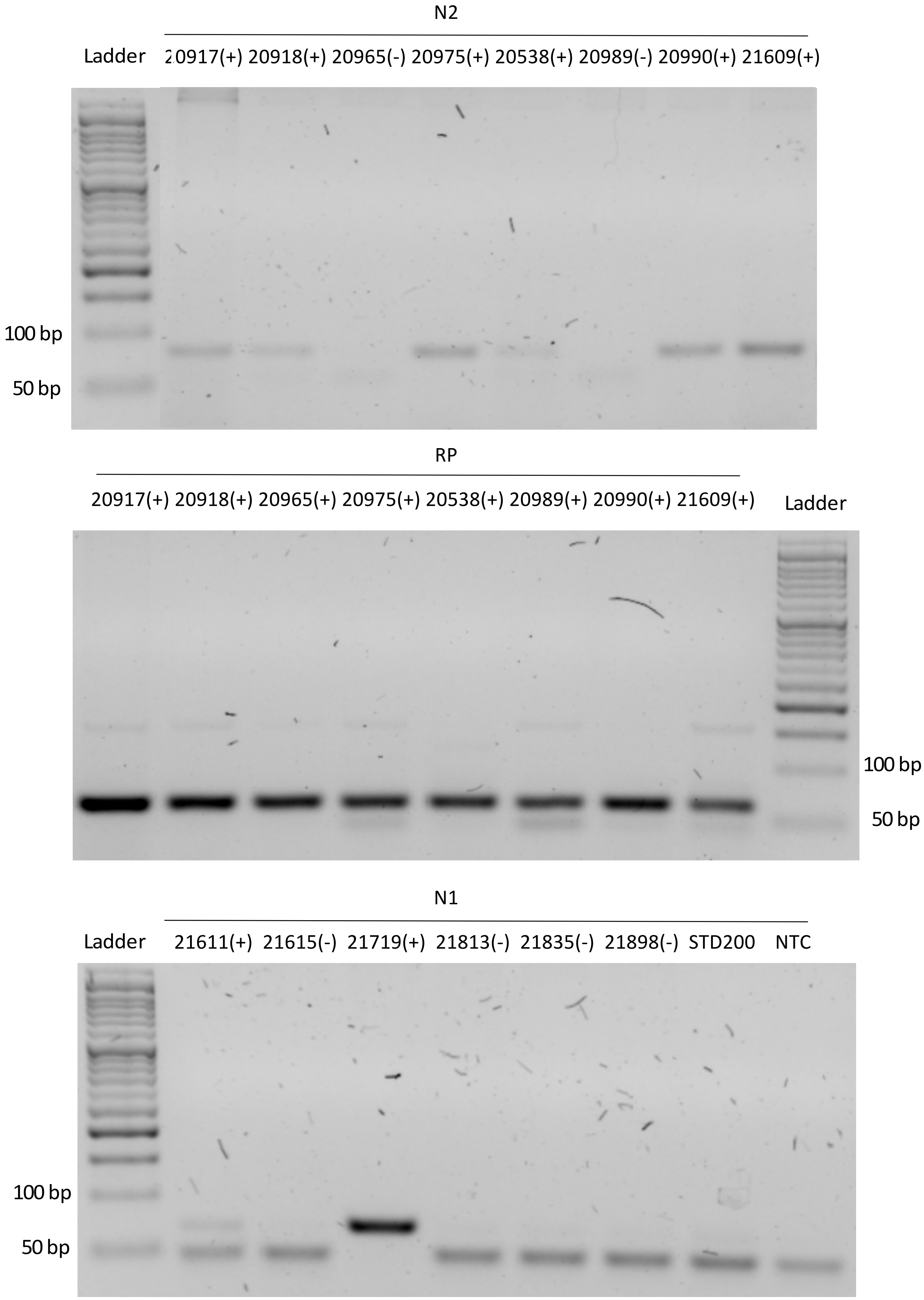
N2
Ladder
20917(+) 20918(+) 20965(-) 20975(+) 20538(+) 20989(-) 20990(+) 21609(+)
100 bp
50 bp
RP
20917(+) 20918(+) 20965(+) 20975(+) 20538(+) 20989(+) 20990(+) 21609(+)
Ladder
100 bp
50 bp
N1
Ladder
21611(+) 21615(-) 21719(+) 21813(-) 21835(-) 21898(-) STD200 NTC
100 bp
50 bp

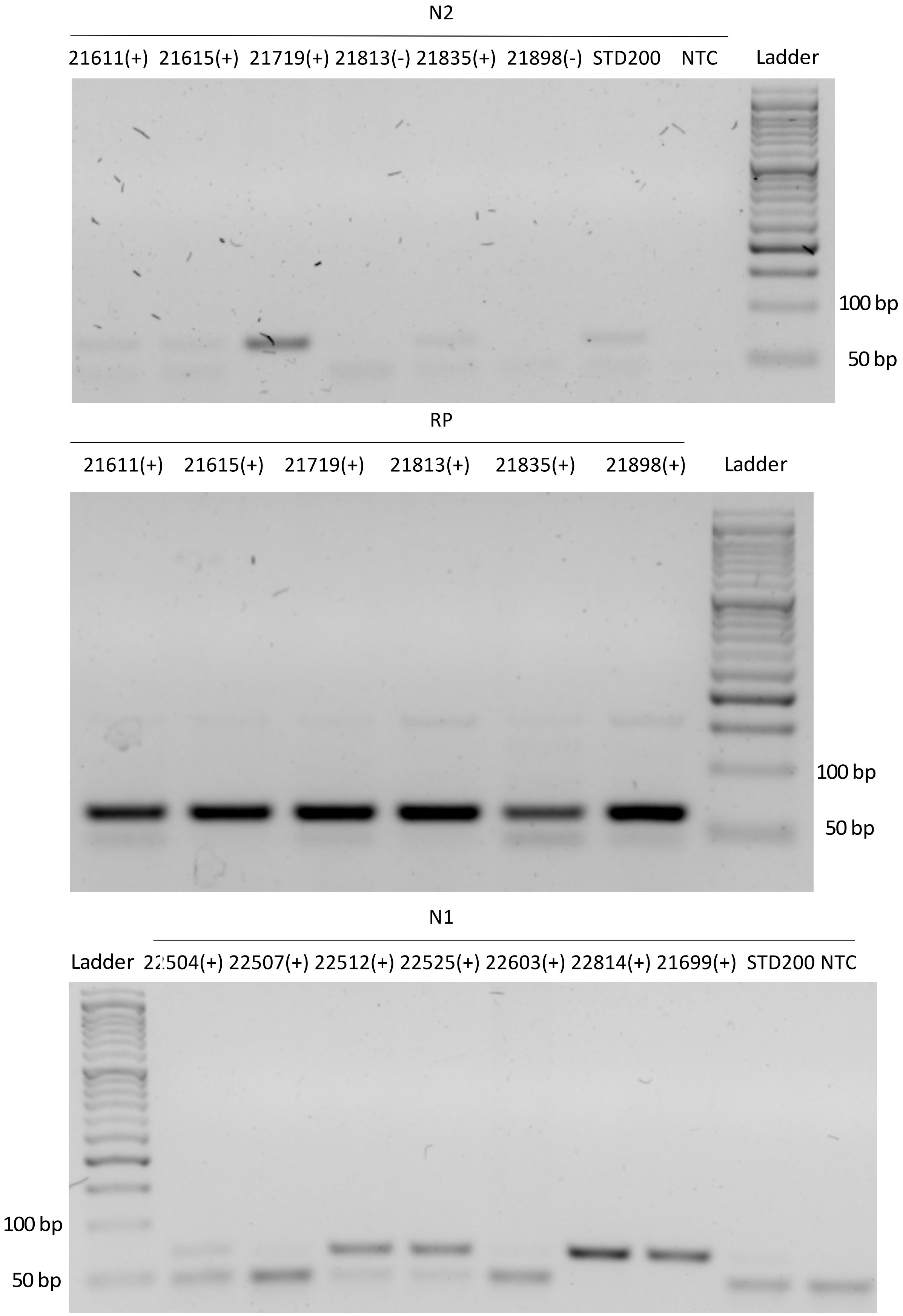
N2
21611(+) 21615(+) 21719(+) 21813(-) 21835(+) 21898(-) STD200 NTC Ladder
100 bp
50 bp
RP
21611(+) 21615(+) 21719(+) 21813(+) 21835(+) 21898(+) Ladder
100 bp
50 bp
N1
Ladder 22504(+) 22507(+) 22512(+) 22525(+) 22603(+) 22814(+) 21699(+) STD200 NTC
100 bp
50 bp

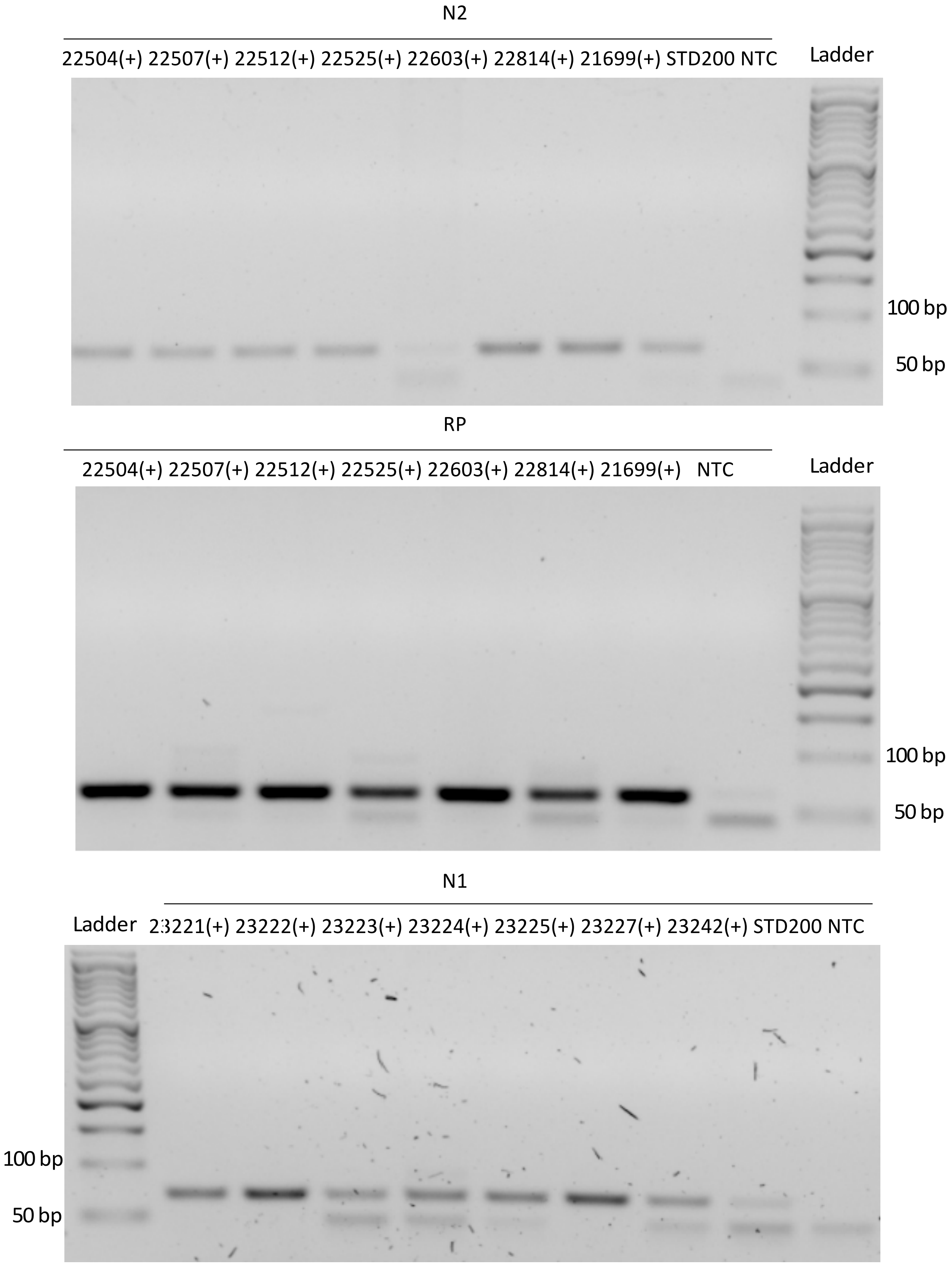
N2
22504(+) 22507(+) 22512(+) 22525(+) 22603(+) 22814(+) 21699(+) STD200 NTC
Ladder
100 bp
50 bp
RP
22504(+) 22507(+) 22512(+) 22525(+) 22603(+) 22814(+) 21699(+) NTC
Ladder
100 bp
50 bp
N1
Ladder
23221(+) 23222(+) 23223(+) 23224(+) 23225(+) 23227(+) 23242(+) STD200 NTC
100 bp
50 bp

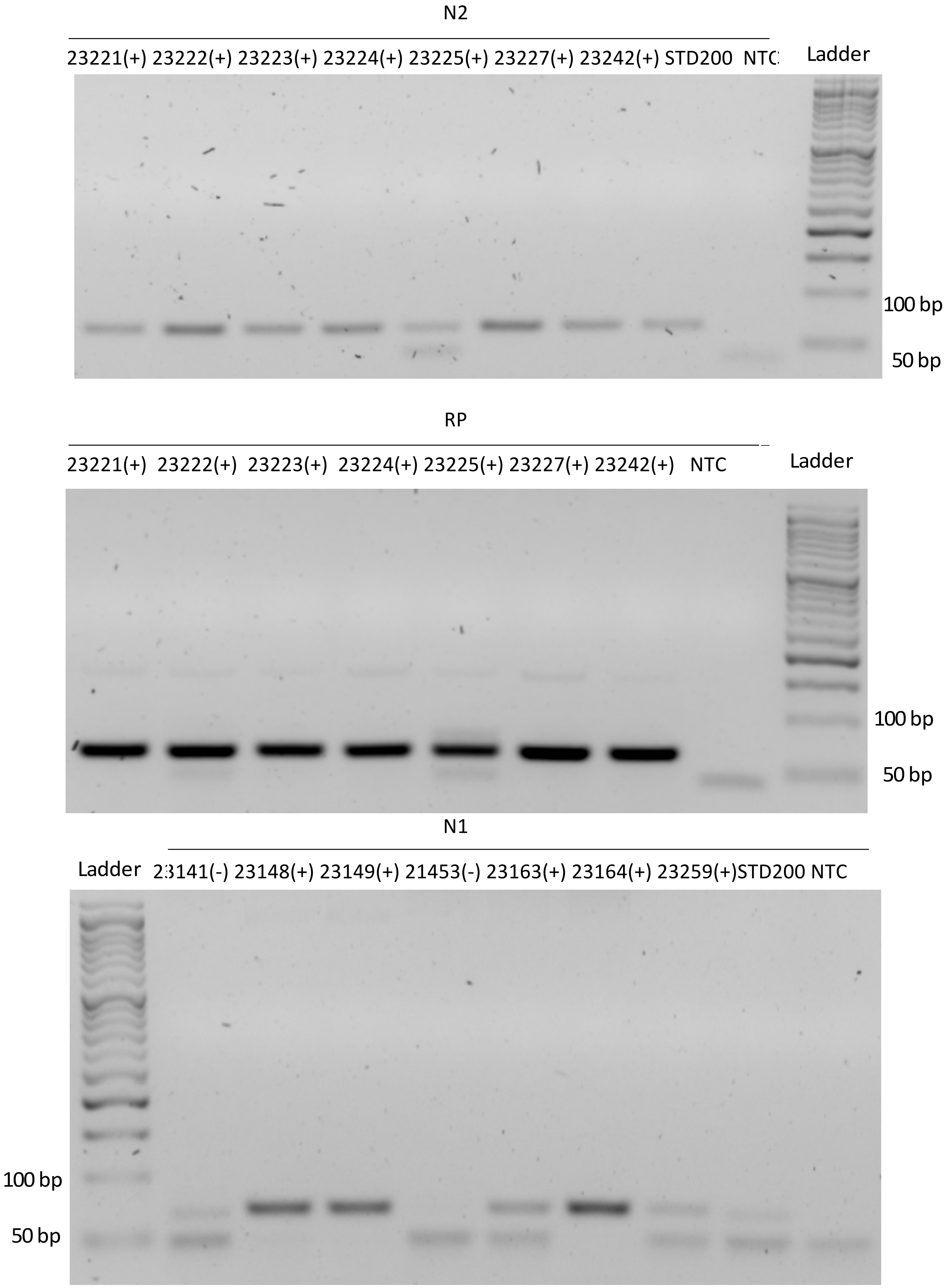
N2
23221(+) 23222(+) 23223(+) 23224(+) 23225(+) 23227(+) 23242(+) STD200 NTC[illegible]
Ladder
100 bp
50 bp
RP
23221(+) 23222(+) 23223(+) 23224(+) 23225(+) 23227(+) 23242(+) NTC
Ladder
100 bp
50 bp
N1
Ladder
23141(-) 23148(+) 23149(+) 21453(-) 23163(+) 23164(+) 23259(+)STD200 NTC
100 bp
50 bp

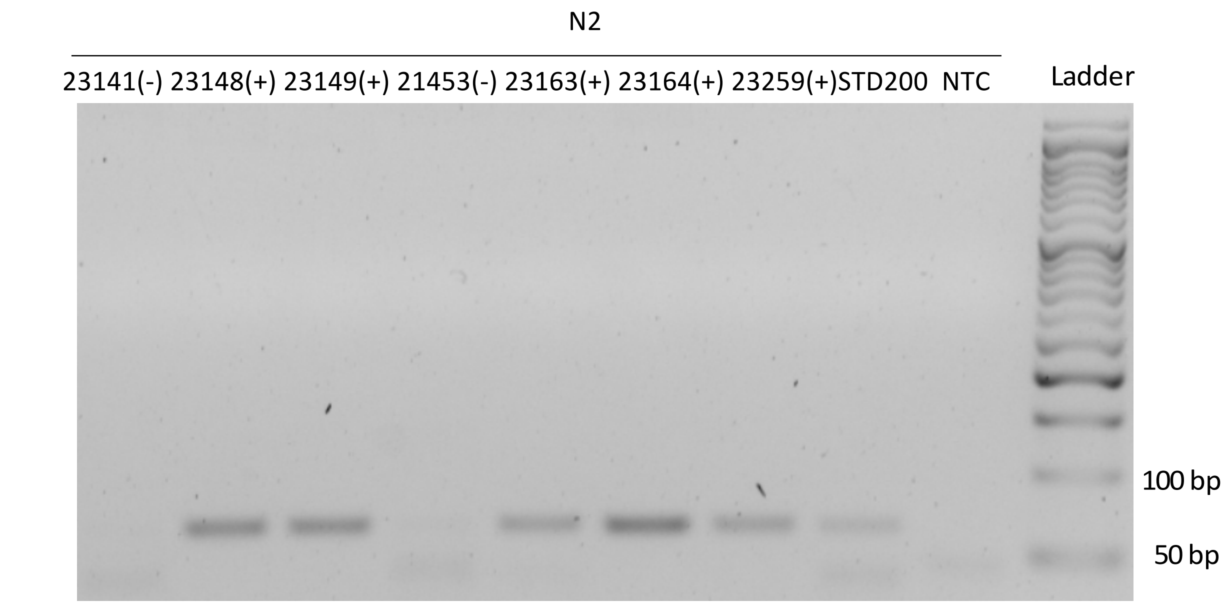
N2
23141(-) 23148(+) 23149(+) 21453(-) 23163(+) 23164(+) 23259(+)STD200 NTC
Ladder
100 bp
50 bp

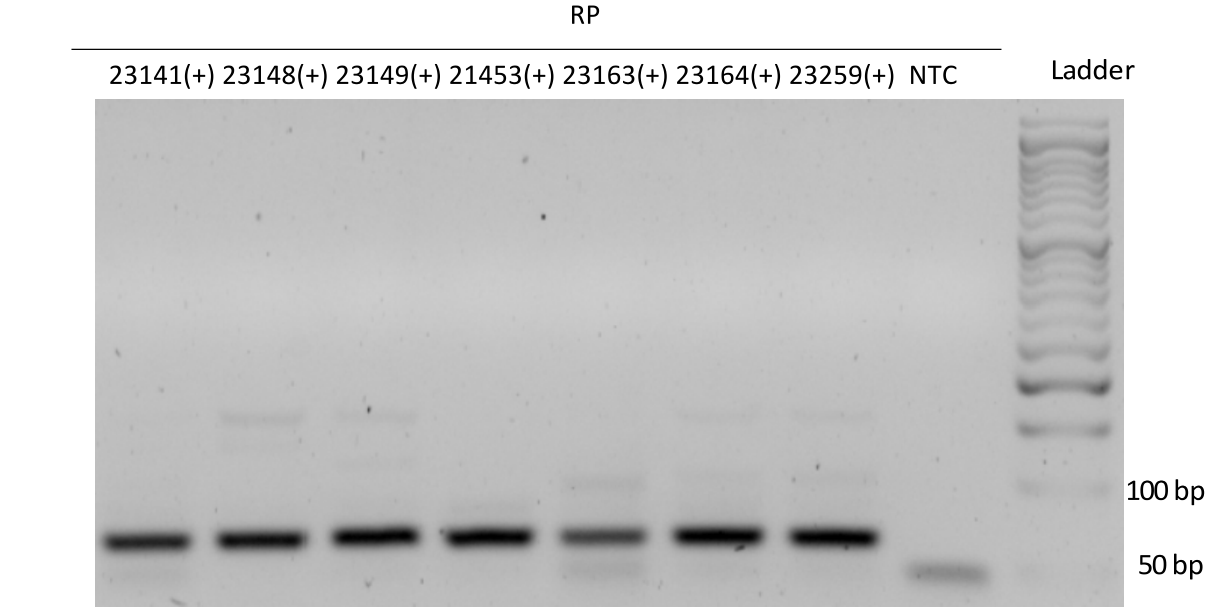
RP
23141(+) 23148(+) 23149(+) 21453(+) 23163(+) 23164(+) 23259(+) NTC
Ladder
100 bp
50 bp

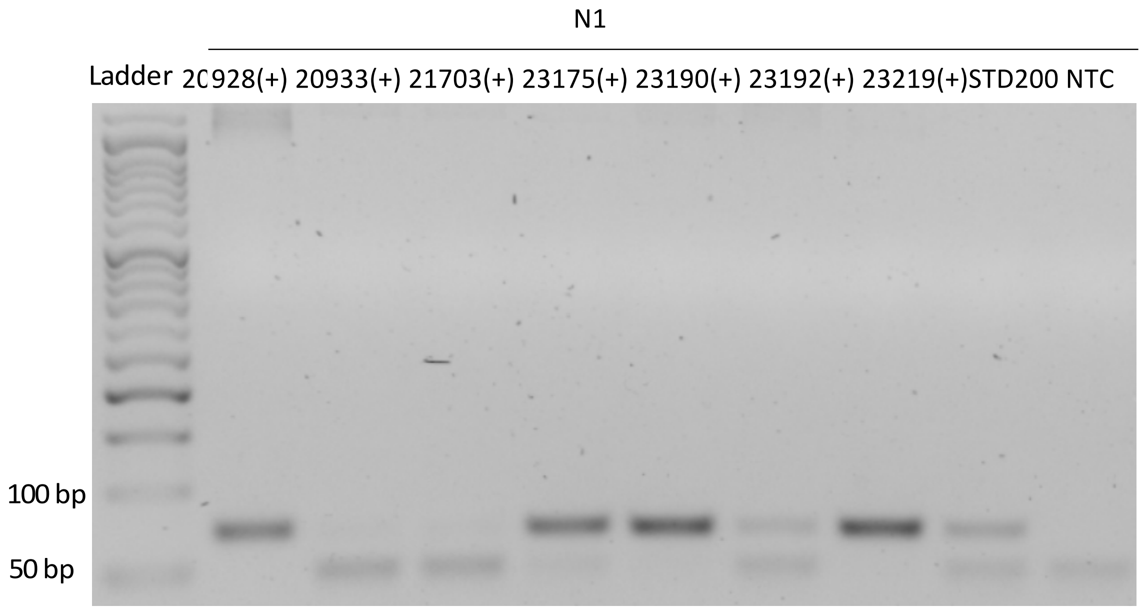
N1
Ladder
2C928(+) 20933(+) 21703(+) 23175(+) 23190(+) 23192(+) 23219(+)STD200 NTC
100 bp
50 bp

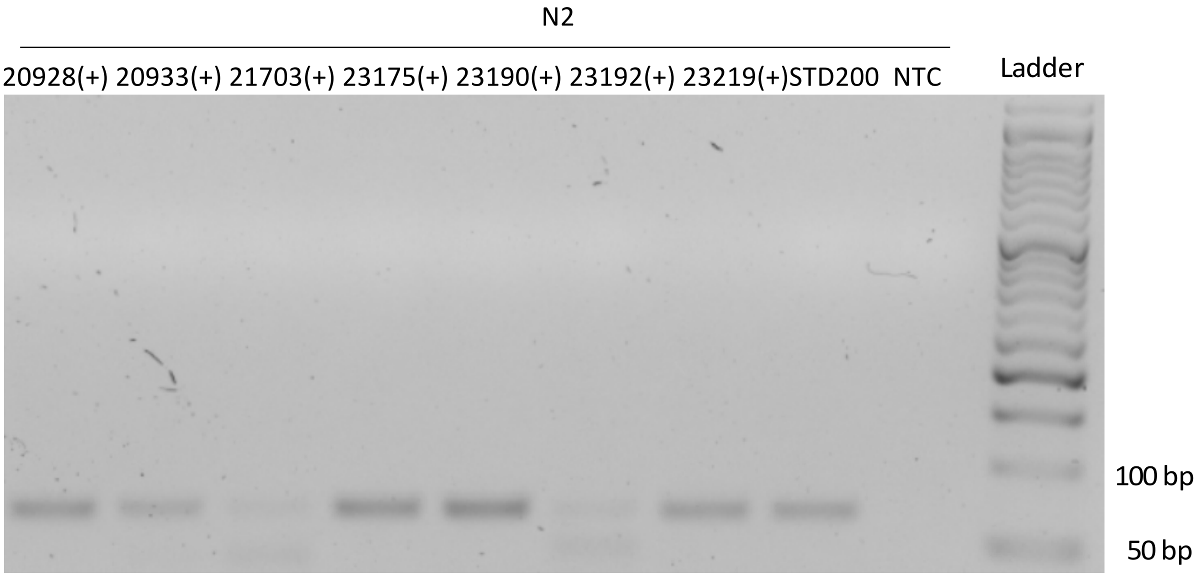
N2
20928(+) 20933(+) 21703(+) 23175(+) 23190(+) 23192(+) 23219(+)STD200 NTC
Ladder
100 bp
50 bp

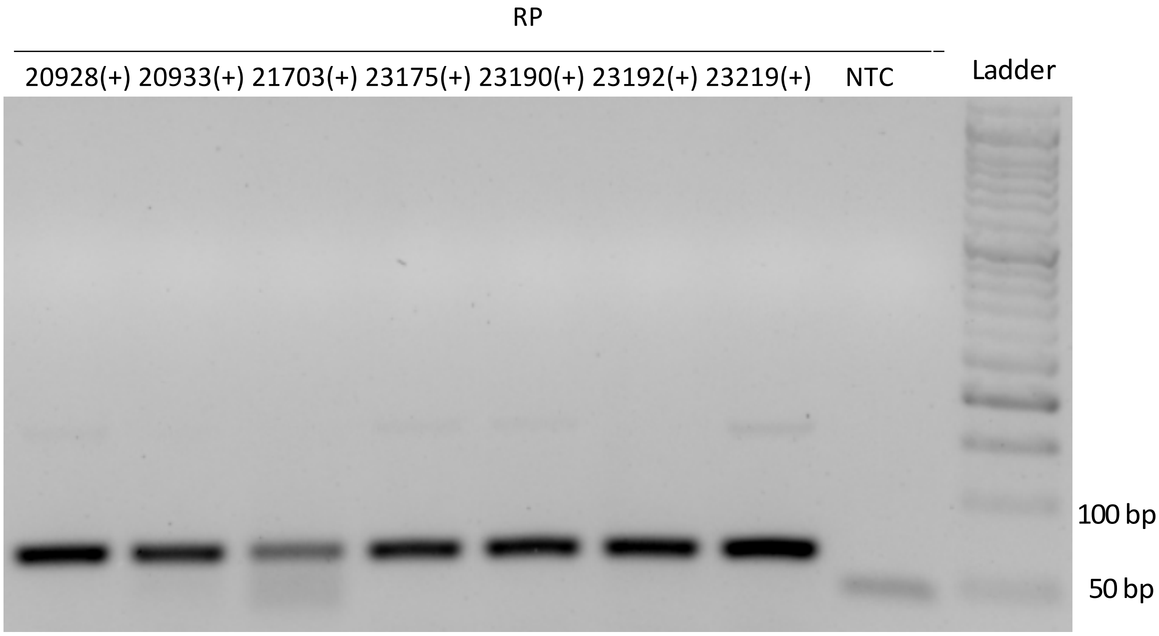
RP
20928(+) 20933(+) 21703(+) 23175(+) 23190(+) 23192(+) 23219(+) NTC
Ladder
100 bp
50 bp

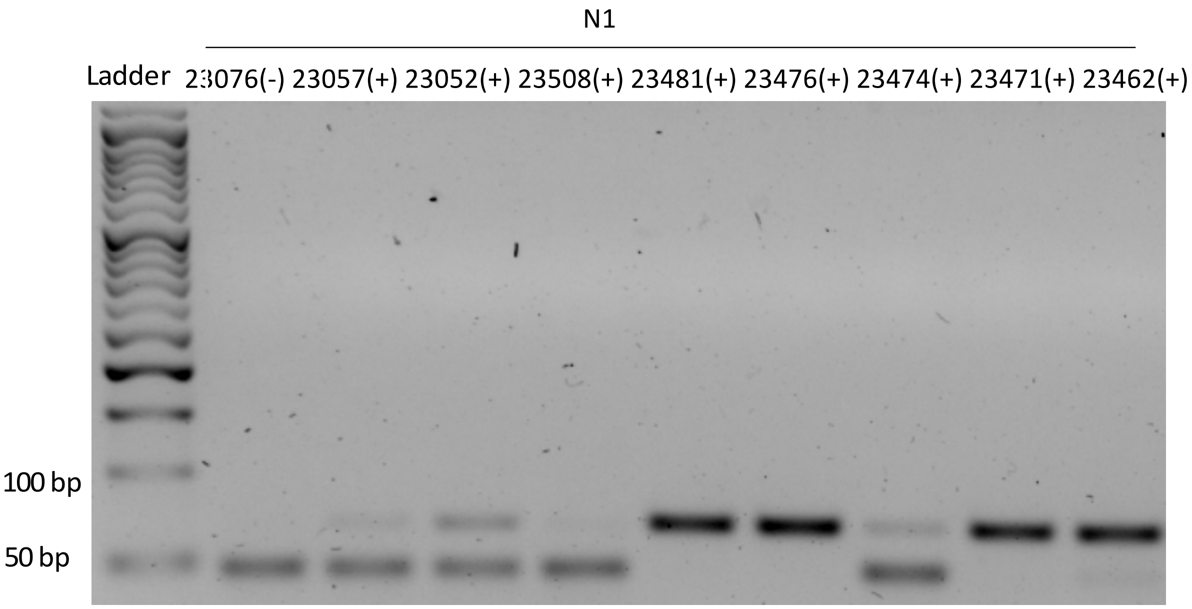
N1
Ladder
23076(-) 23057(+) 23052(+) 23508(+) 23481(+) 23476(+) 23474(+) 23471(+) 23462(+)
100 bp
50 bp

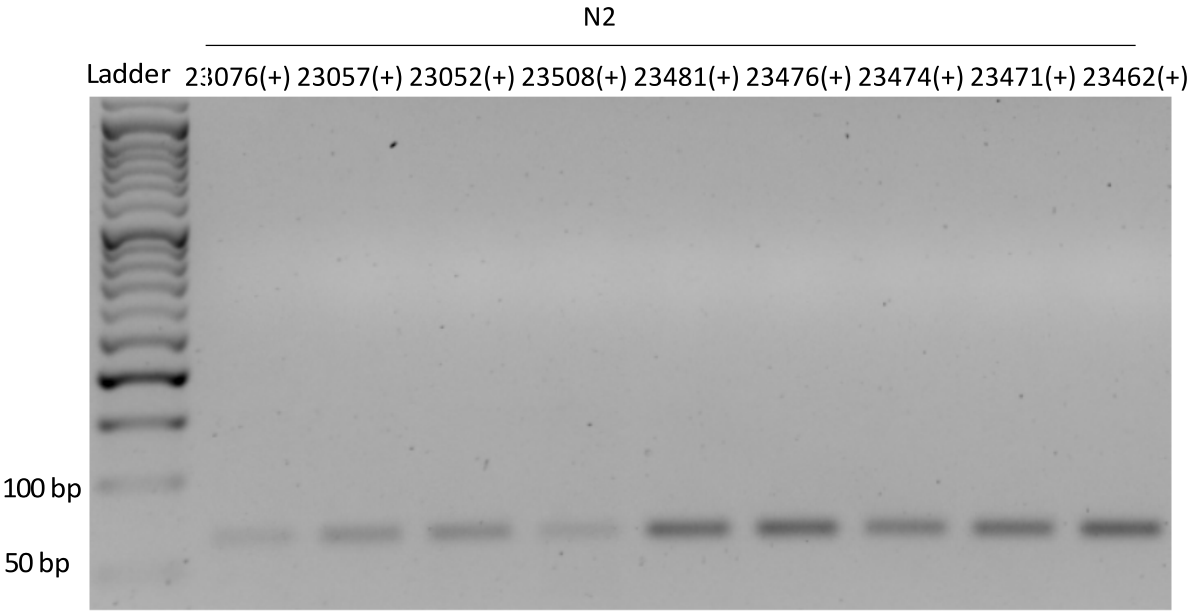
N2
Ladder
23076(+) 23057(+) 23052(+) 23508(+) 23481(+) 23476(+) 23474(+) 23471(+) 23462(+)
100 bp
50 bp

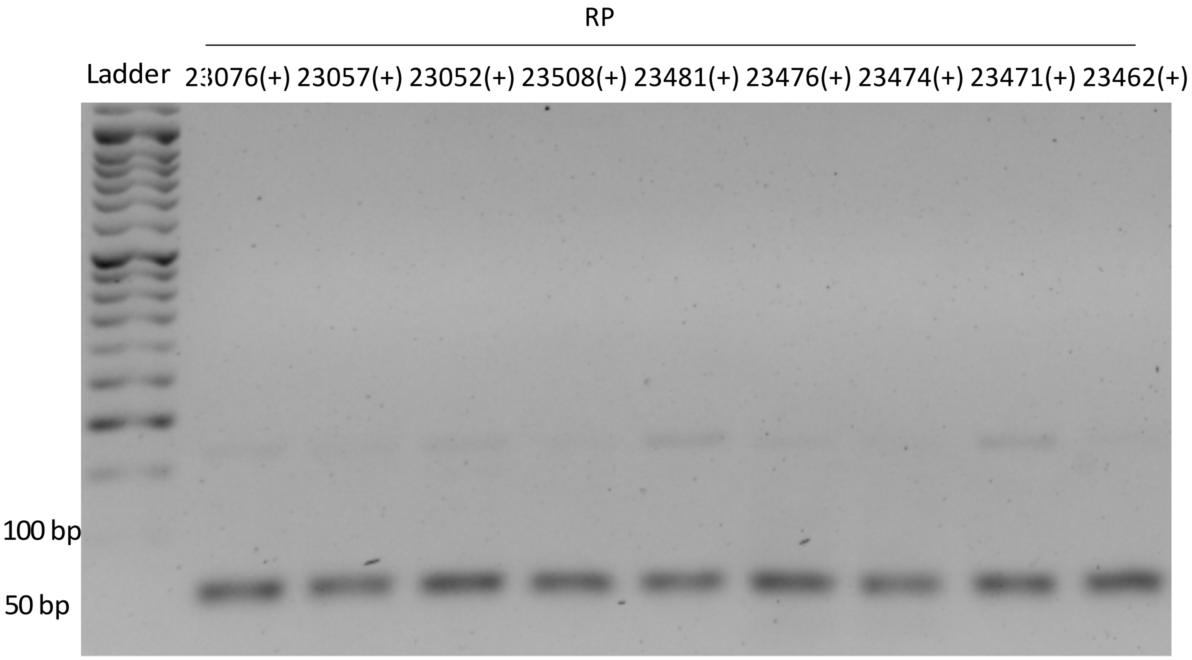
RP
Ladder
23076(+) 23057(+) 23052(+) 23508(+) 23481(+) 23476(+) 23474(+) 23471(+) 23462(+)
100 bp
50 bp

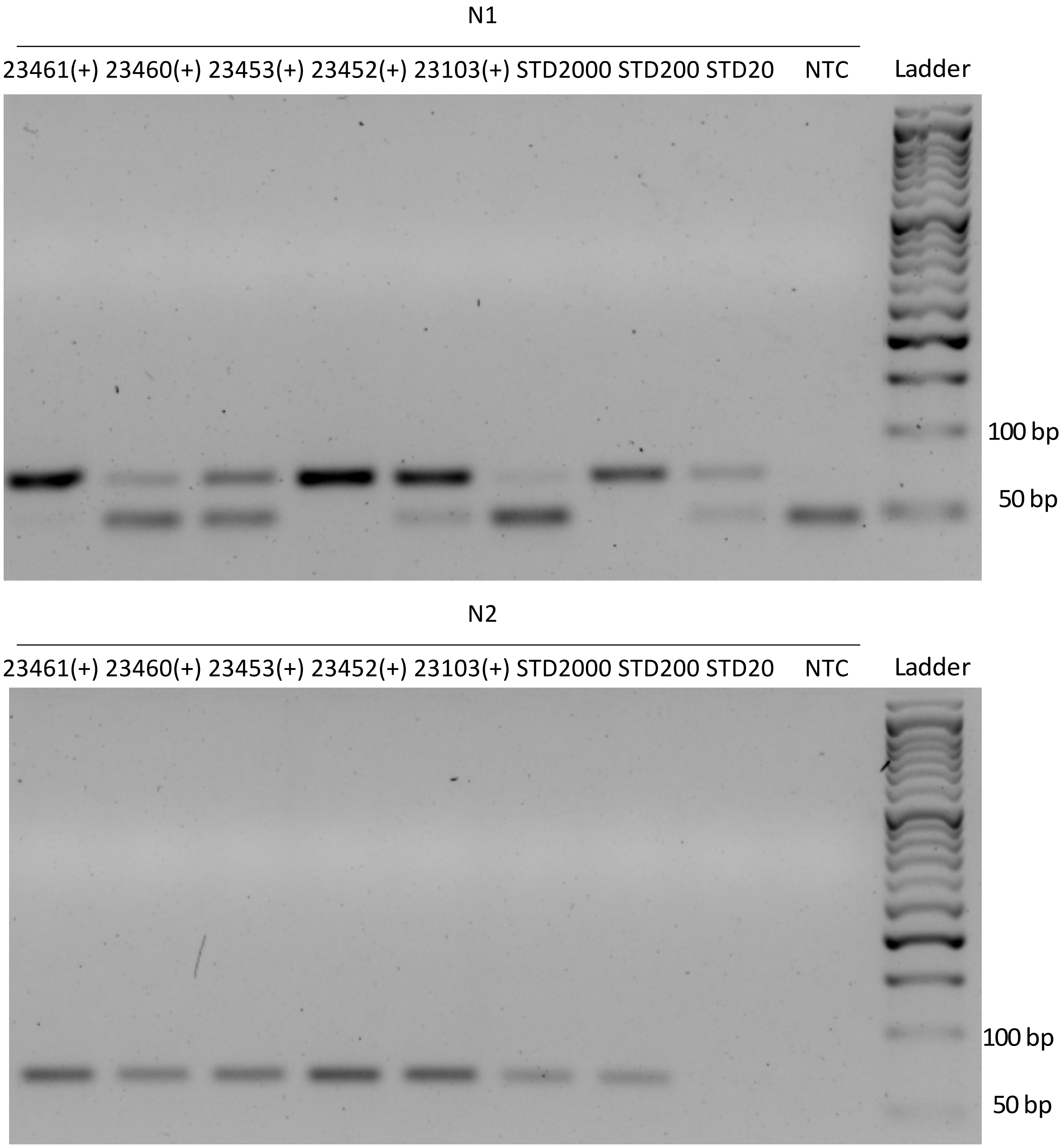
N1
23461(+) 23460(+) 23453(+) 23452(+) 23103(+) STD2000 STD200 STD20 NTC Ladder
100 bp
50 bp
N2
23461(+) 23460(+) 23453(+) 23452(+) 23103(+) STD2000 STD200 STD20 NTC Ladder
100 bp
50 bp

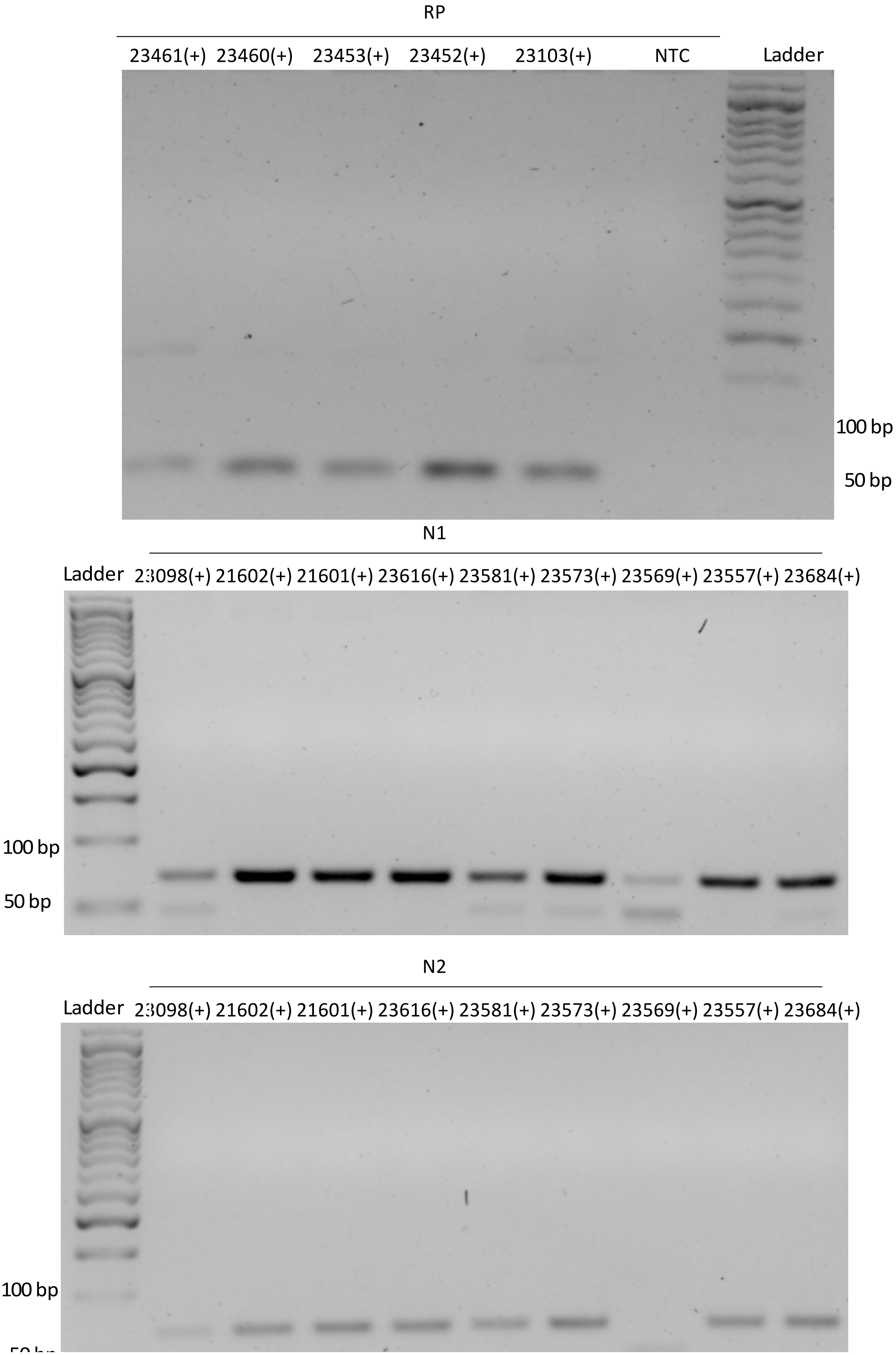

RP
23461(+) 23460(+) 23453(+) 23452(+) 23103(+) NTC Ladder
100 bp
50 bp
N1
Ladder 23098(+) 21602(+) 21601(+) 23616(+) 23581(+) 23573(+) 23569(+) 23557(+) 23684(+)
100 bp
50 bp
N2
Ladder 23098(+) 21602(+) 21601(+) 23616(+) 23581(+) 23573(+) 23569(+) 23557(+) 23684(+)
100 bp

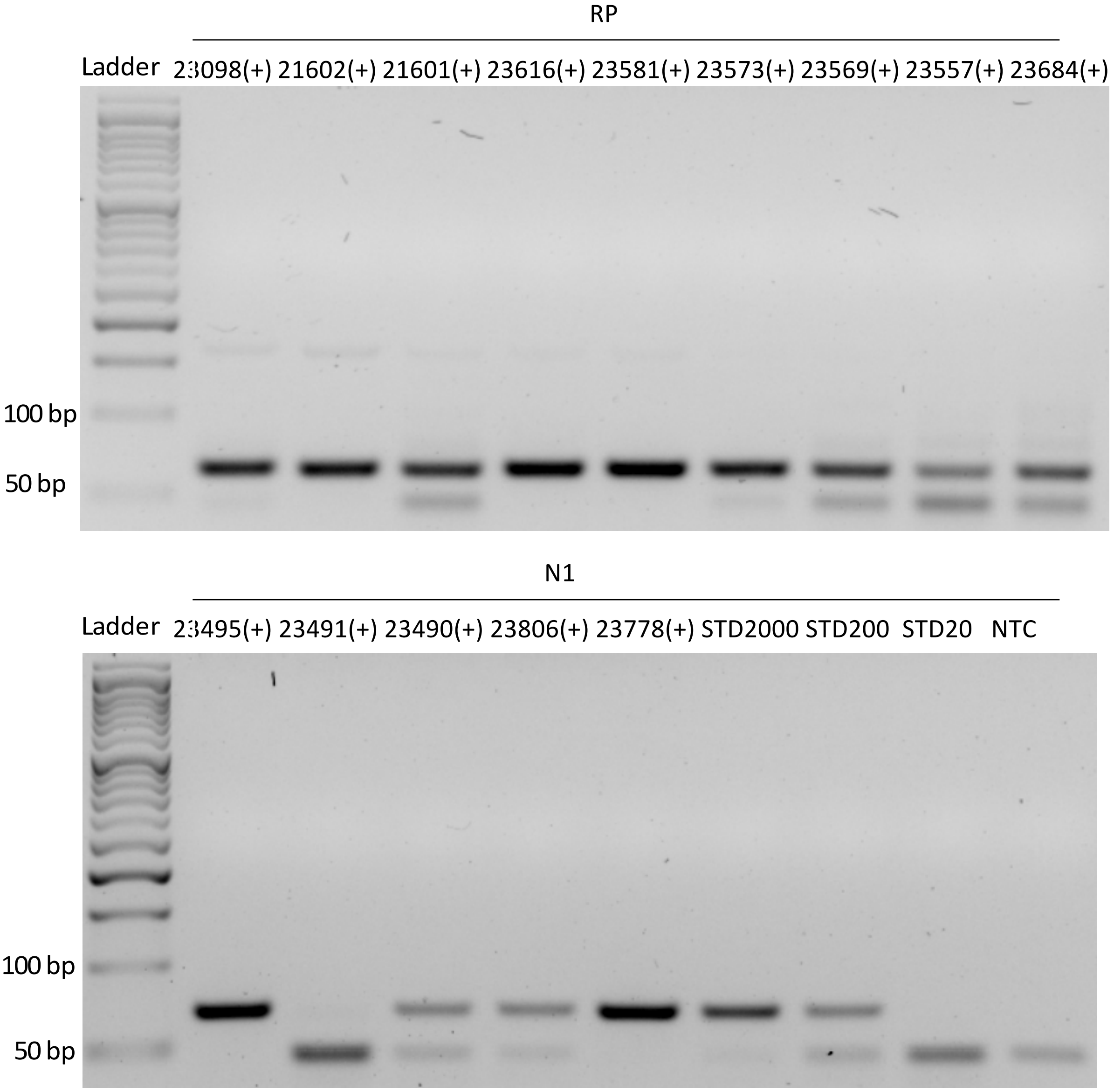

RP
Ladder
23098(+)
21602(+)
21601(+)
23616(+)
23581(+)
23573(+)
23569(+)
23557(+)
23684(+)
100 bp
50 bp
N1
Ladder
23495(+)
23491(+)
23490(+)
23806(+)
23778(+)
STD2000
STD200
STD20
NTC
100 bp
50 bp

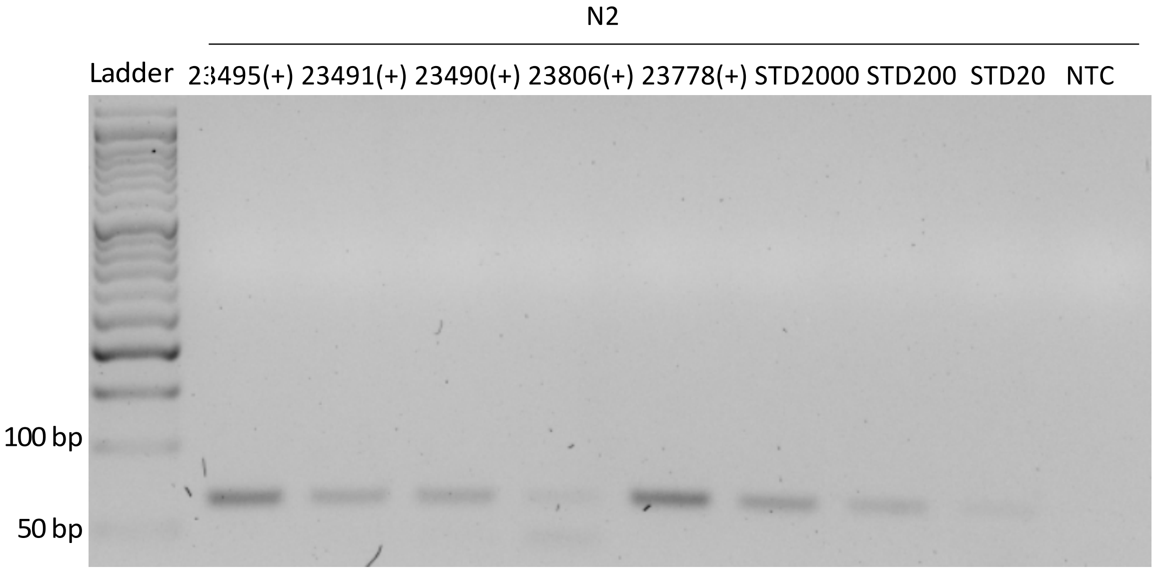
N2
Ladder
23495(+)
23491(+)
23490(+)
23806(+)
23778(+)
STD2000
STD200
STD20
NTC
100 bp
50 bp

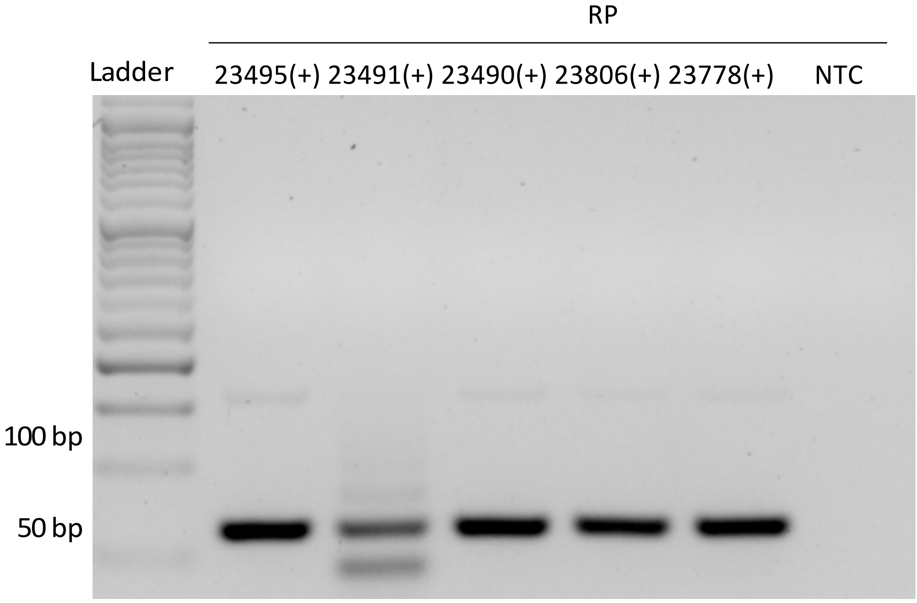
RP
Ladder
23495(+)
23491(+)
23490(+)
23806(+)
23778(+)
NTC
100 bp
50 bp

SARS-CoV-2 Positive and Negative Samples

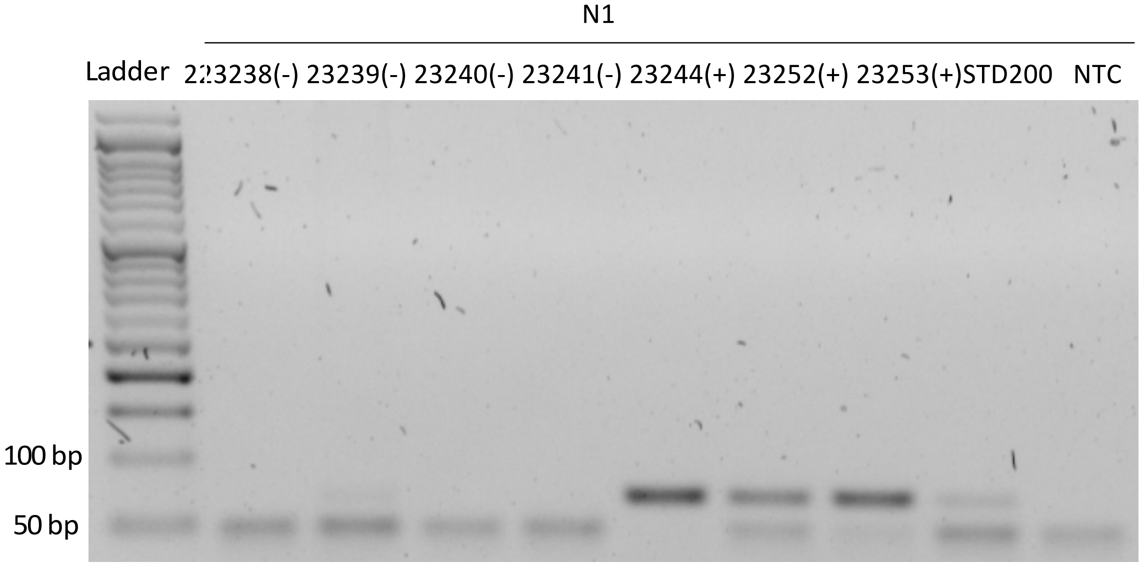

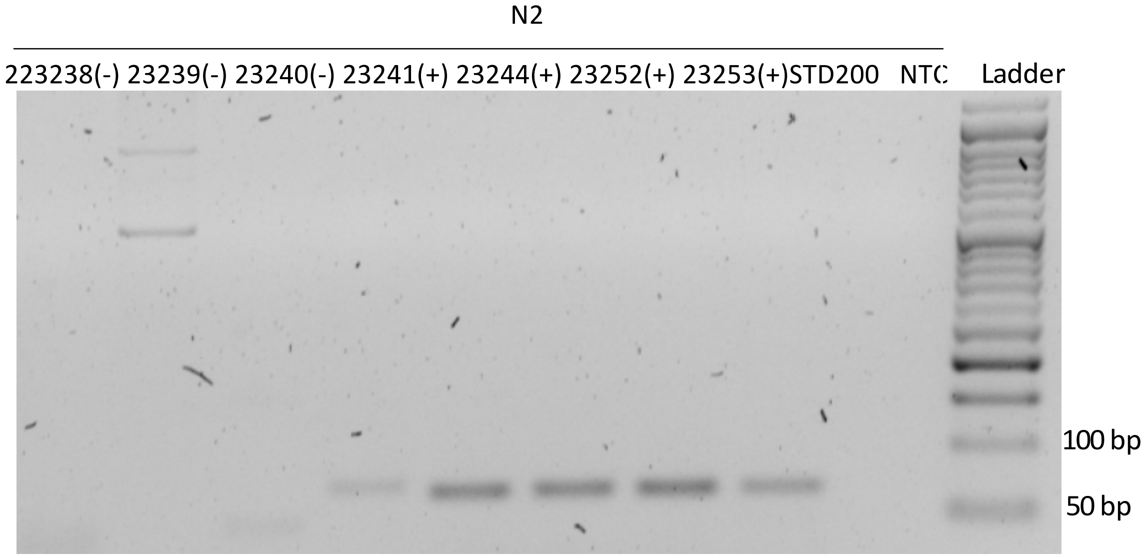

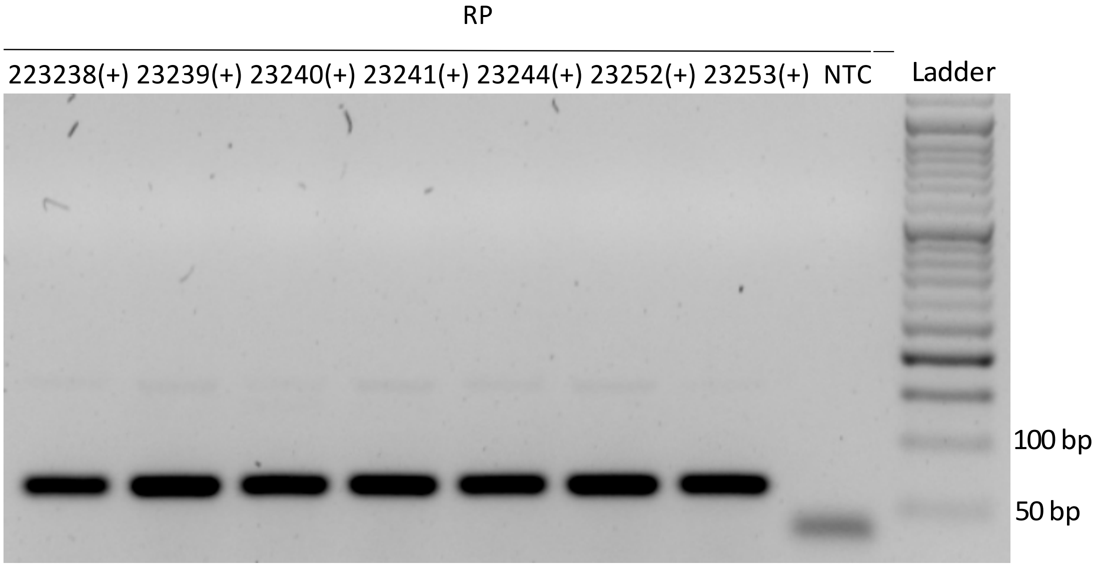

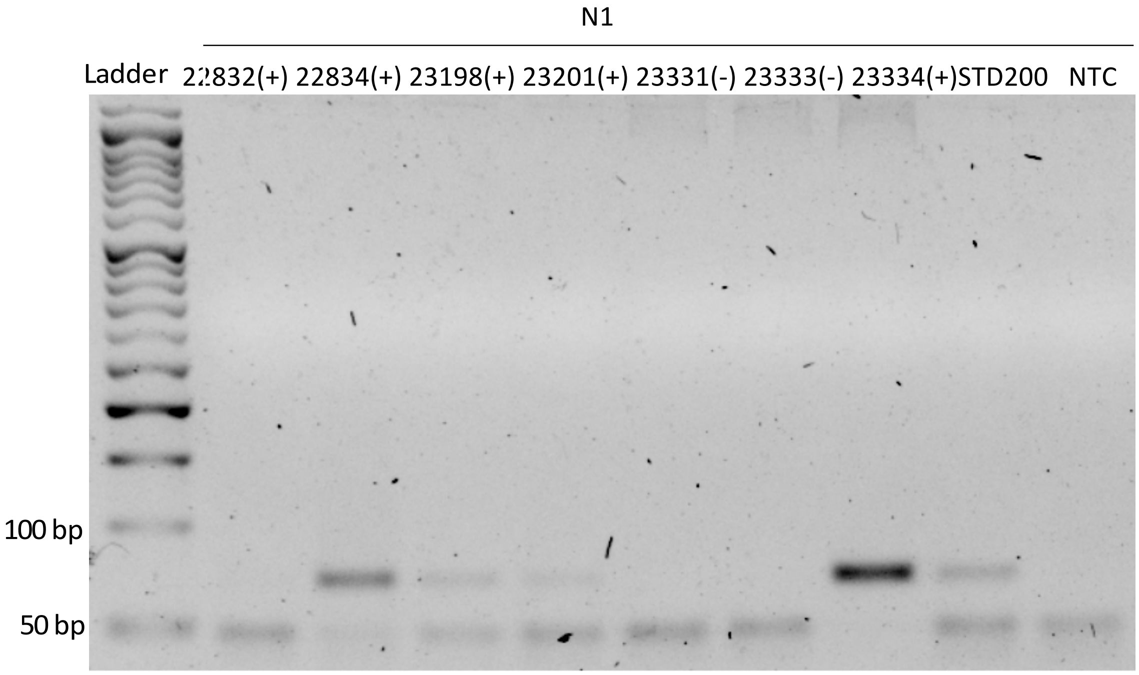
N1
Ladder 22832(+) 22834(+) 23198(+) 23201(+) 23331(-) 23333(-) 23334(+) STD200 NTC
100 bp
50 bp

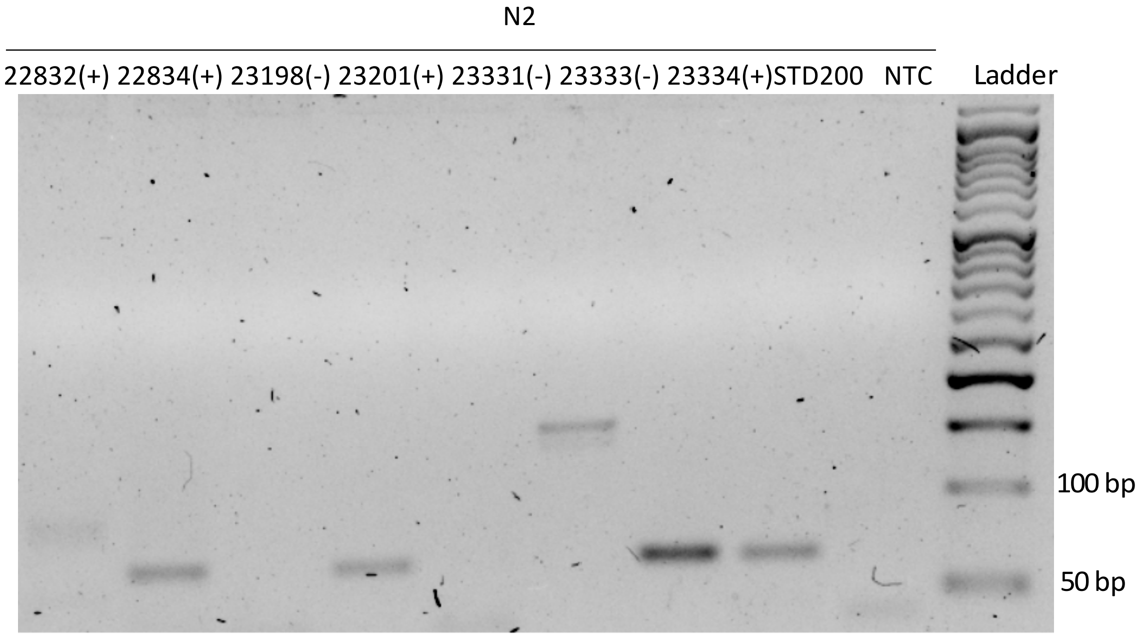
N2
22832(+) 22834(+) 23198(-) 23201(+) 23331(-) 23333(-) 23334(+) STD200 NTC Ladder
100 bp
50 bp

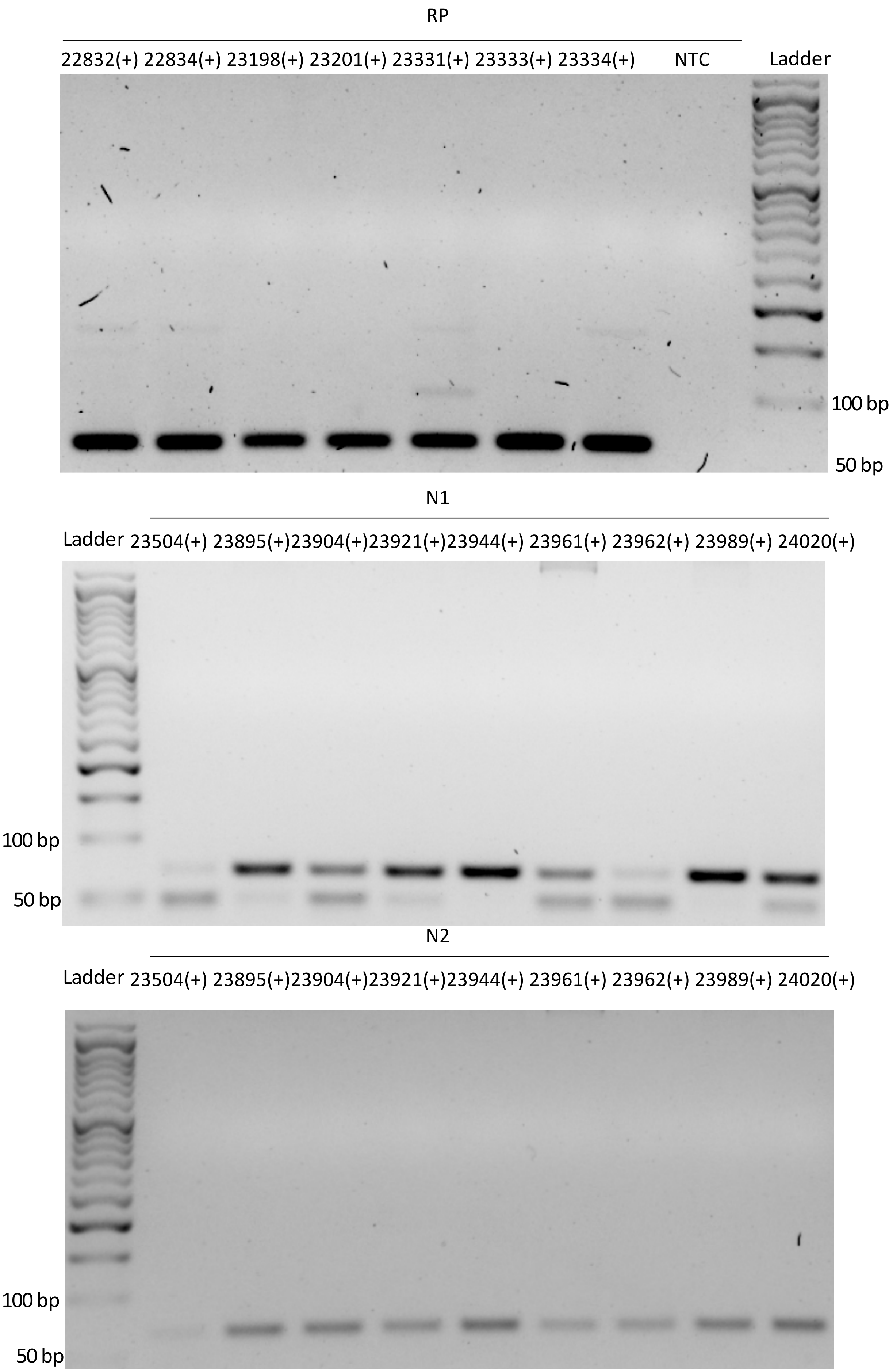
RP
22832(+) 22834(+) 23198(+) 23201(+) 23331(+) 23333(+) 23334(+) NTC Ladder
100 bp
50 bp
N1
Ladder 23504(+) 23895(+)23904(+)23921(+)23944(+) 23961(+) 23962(+) 23989(+) 24020(+)
100 bp
50 bp
N2
Ladder 23504(+) 23895(+)23904(+)23921(+)23944(+) 23961(+) 23962(+) 23989(+) 24020(+)
100 bp
50 bp

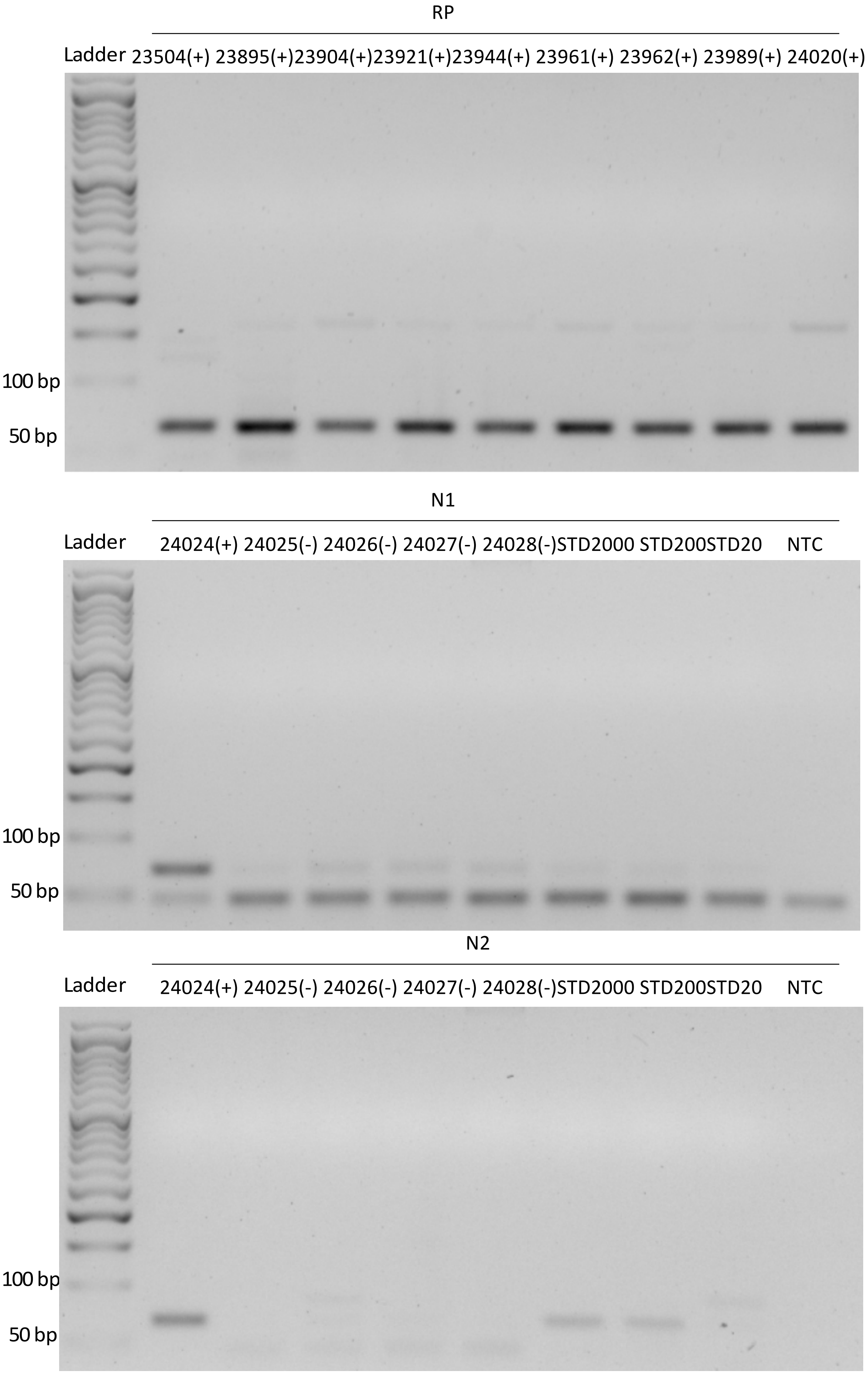
RP
Ladder
23504(+) 23895(+) 23904(+) 23921(+) 23944(+) 23961(+) 23962(+) 23989(+) 24020(+)
100 bp
50 bp
N1
Ladder
24024(+) 24025(-) 24026(-) 24027(-) 24028(-) STD2000 STD200 STD20 NTC
100 bp
50 bp
N2
Ladder
24024(+) 24025(-) 24026(-) 24027(-) 24028(-) STD2000 STD200 STD20 NTC
100 bp
50 bp

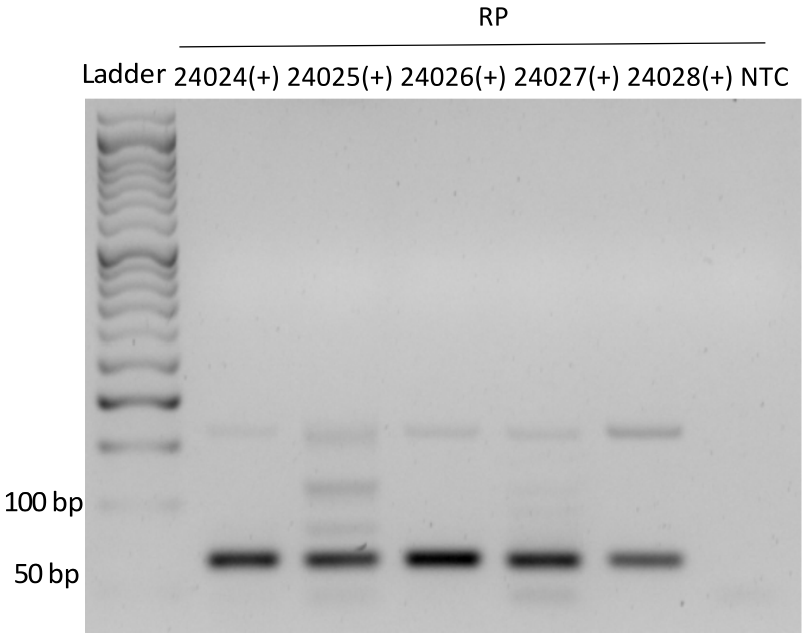
RP
Ladder 24024(+) 24025(+) 24026(+) 24027(+) 24028(+) NTC
100 bp
50 bp

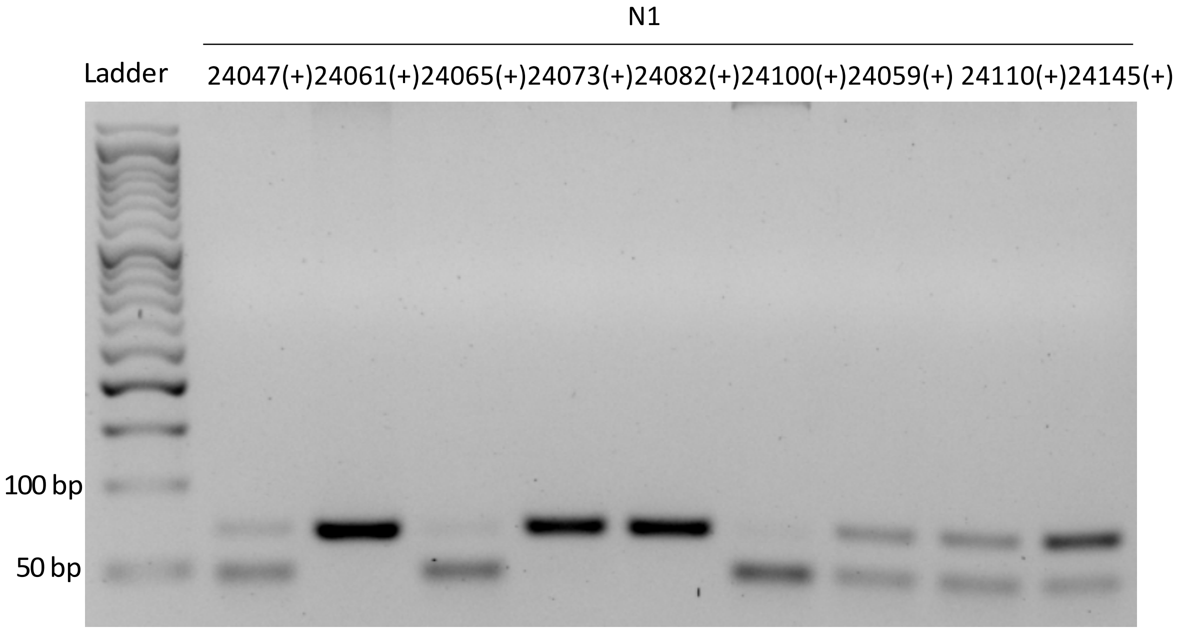
N1
Ladder 24047(+) 24061(+) 24065(+) 24073(+) 24082(+) 24100(+) 24059(+) 24110(+) 24145(+)
100 bp
50 bp

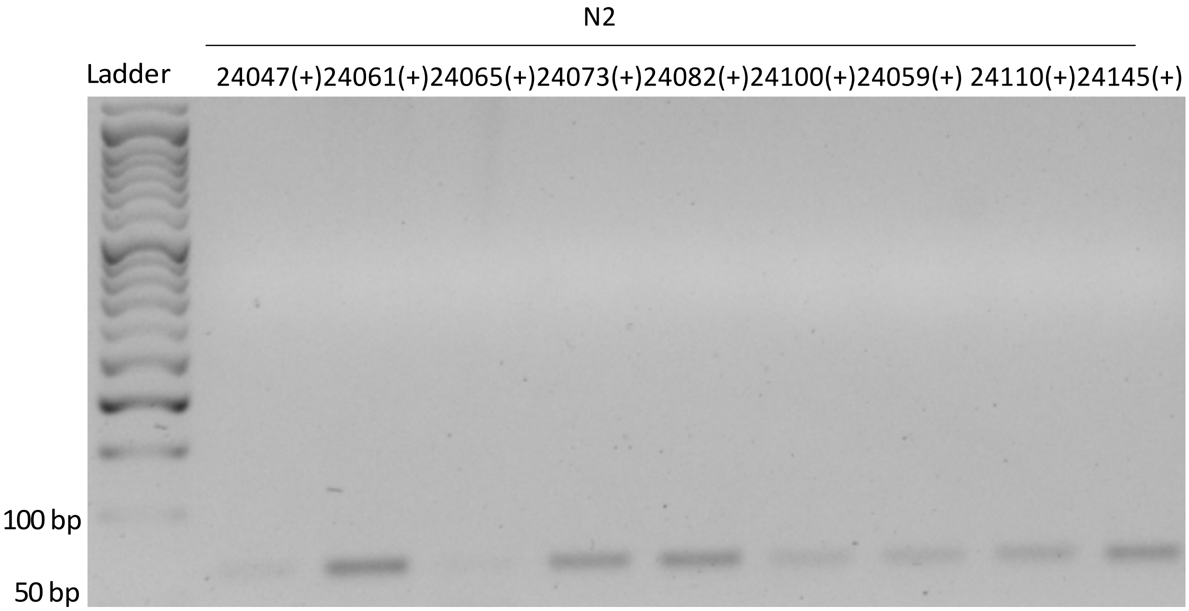
N2
Ladder
24047(+)
24061(+)
24065(+)
24073(+)
24082(+)
24100(+)
24059(+)
24110(+)
24145(+)
100 bp
50 bp

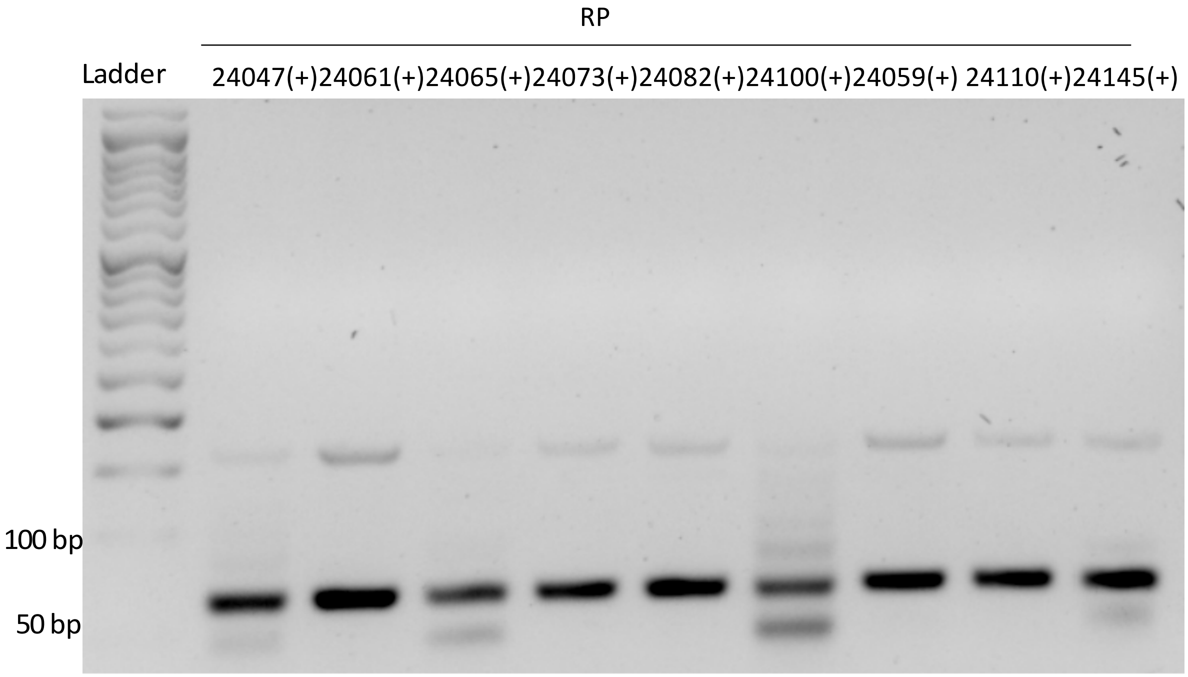
RP
Ladder
24047(+)
24061(+)
24065(+)
24073(+)
24082(+)
24100(+)
24059(+)
24110(+)
24145(+)
100 bp
50 bp

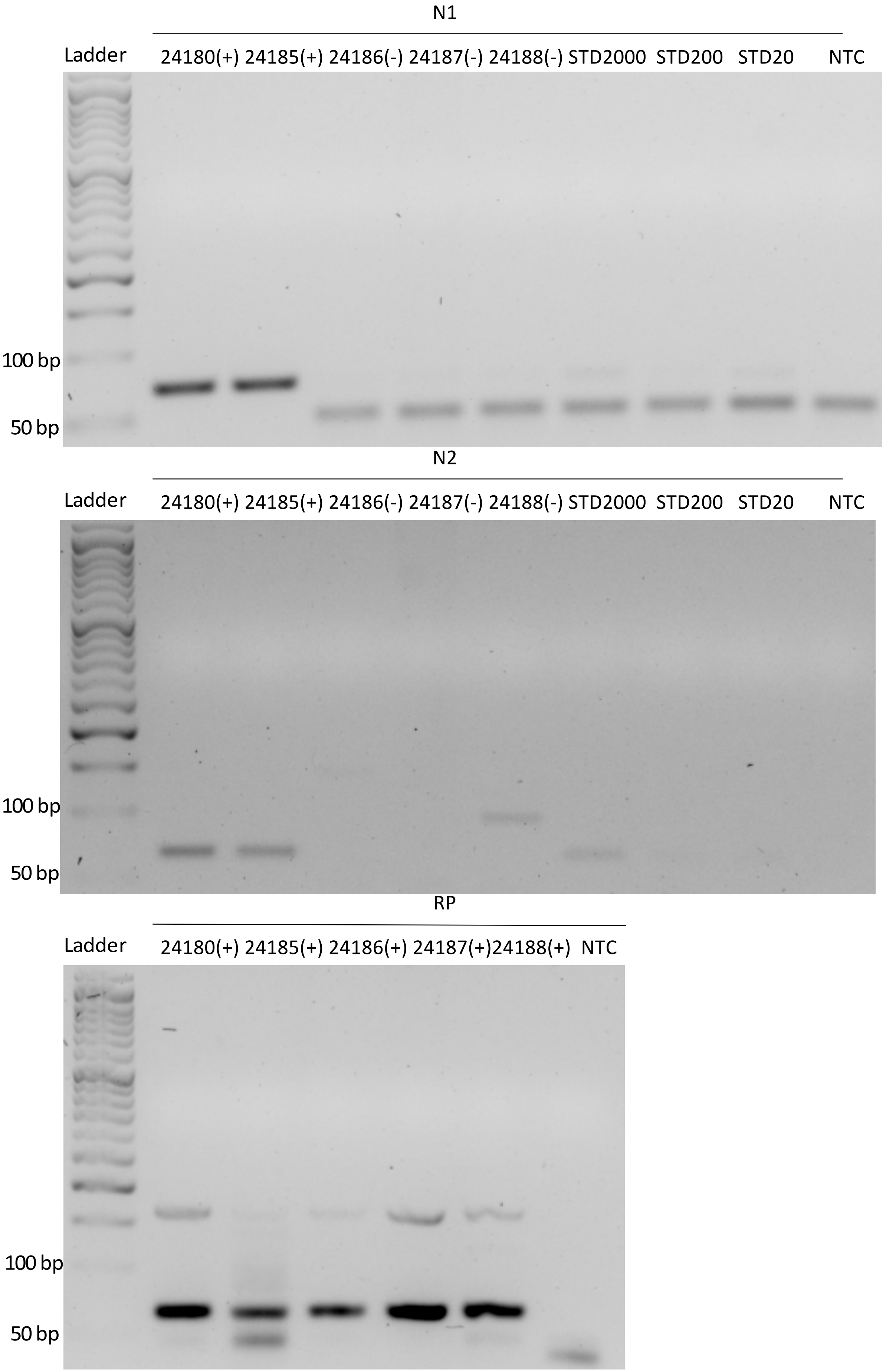
N1
Ladder
24180(+) 24185(+) 24186(-) 24187(-) 24188(-) STD2000 STD200 STD20 NTC
100 bp
50 bp
N2
Ladder
24180(+) 24185(+) 24186(-) 24187(-) 24188(-) STD2000 STD200 STD20 NTC
100 bp
50 bp
RP
Ladder
24180(+) 24185(+) 24186(+) 24187(+)24188(+) NTC
100 bp
50 bp

SARS-CoV-2 Negative Samples

N1

Ladder 23156(-) 23157(-) 23158(-) 23159(-) 23160(-) 23161(-) 23162(-) STD200 NTC

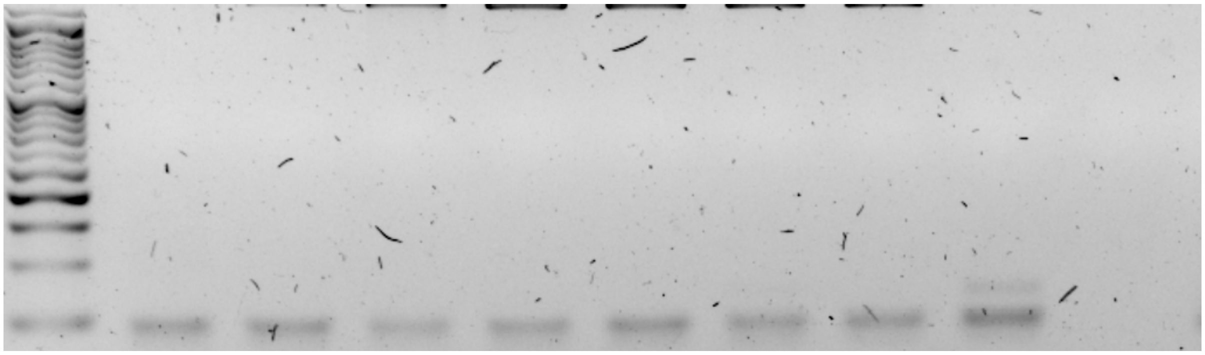

100 bp
50 bp

N2

Ladder 23156(-) 23157(-) 23158(-) 23159(-) STD200 NTC 23160(-) 23161(-) 23162(-)

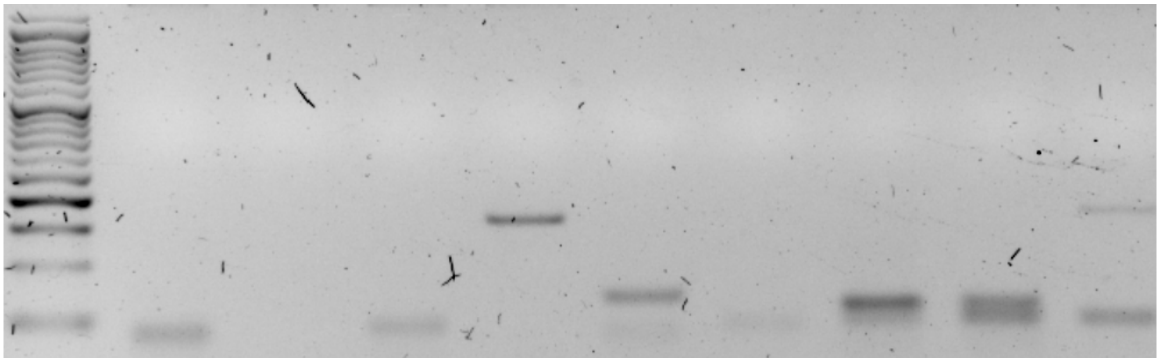

100 bp
50 bp

RP

23156(+) 23157(+) 23158(+) 23159(+) 23160(+) 23161(+) 23162(+) NTC Ladder

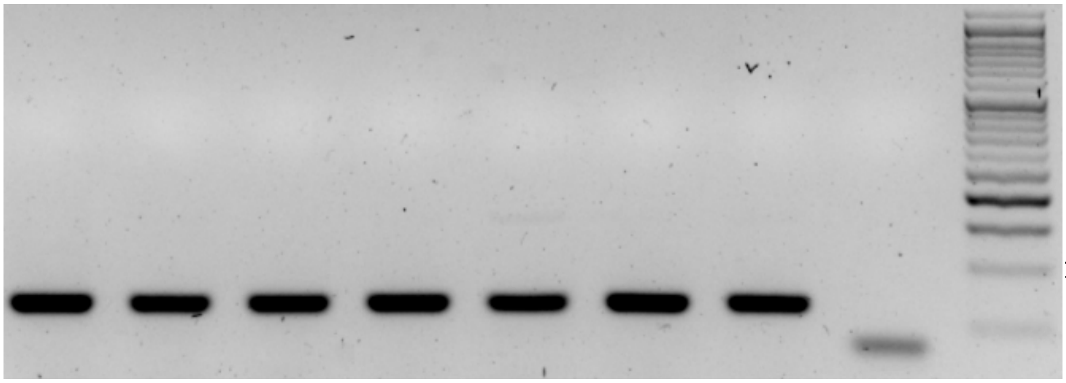

100 bp
50 bp

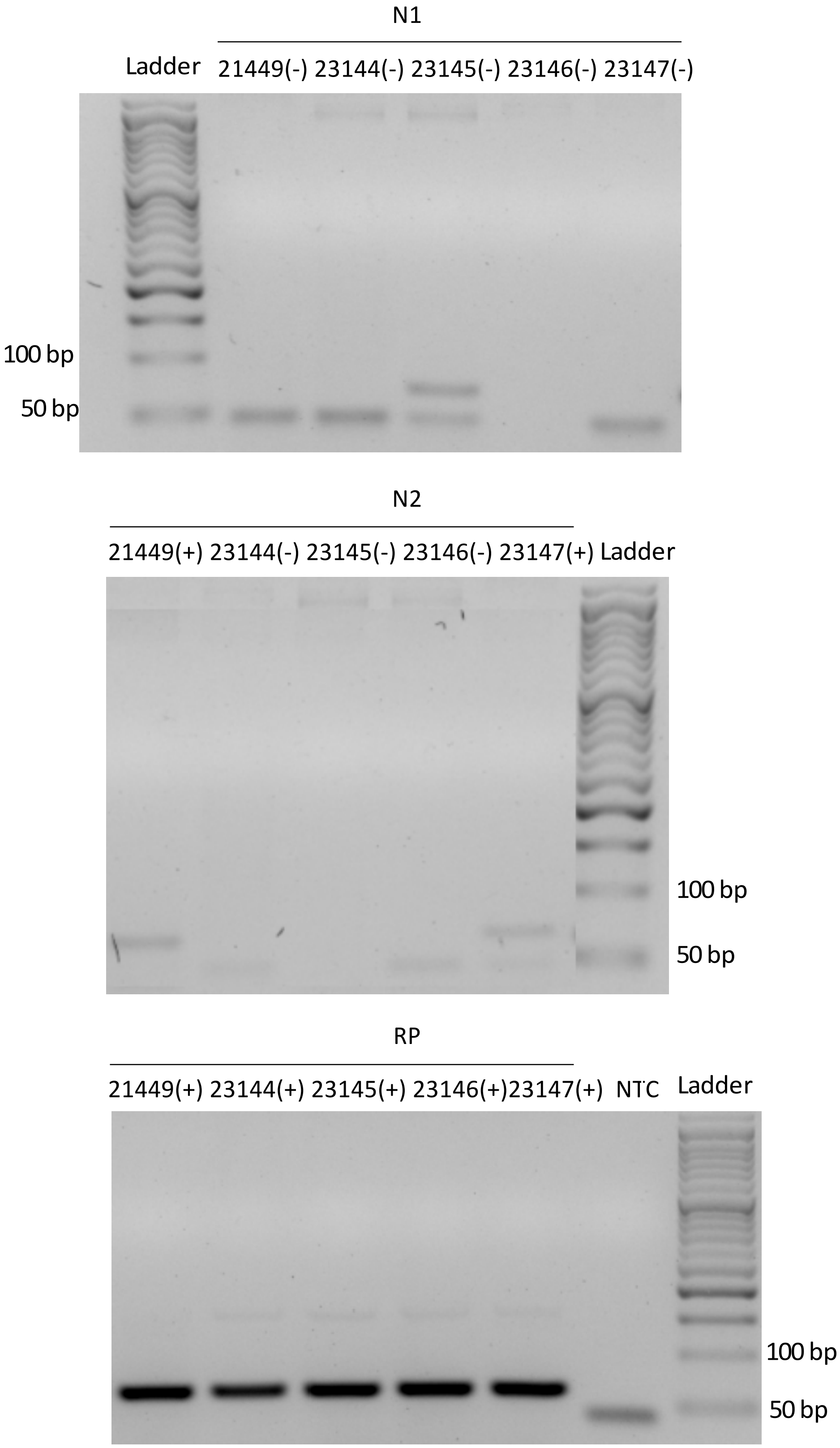
N1
Ladder
21449(-) 23144(-) 23145(-) 23146(-) 23147(-)
100 bp
50 bp
N2
21449(+) 23144(-) 23145(-) 23146(-) 23147(+) Ladder
100 bp
50 bp
RP
21449(+) 23144(+) 23145(+) 23146(+)23147(+) NTC Ladder
100 bp
50 bp

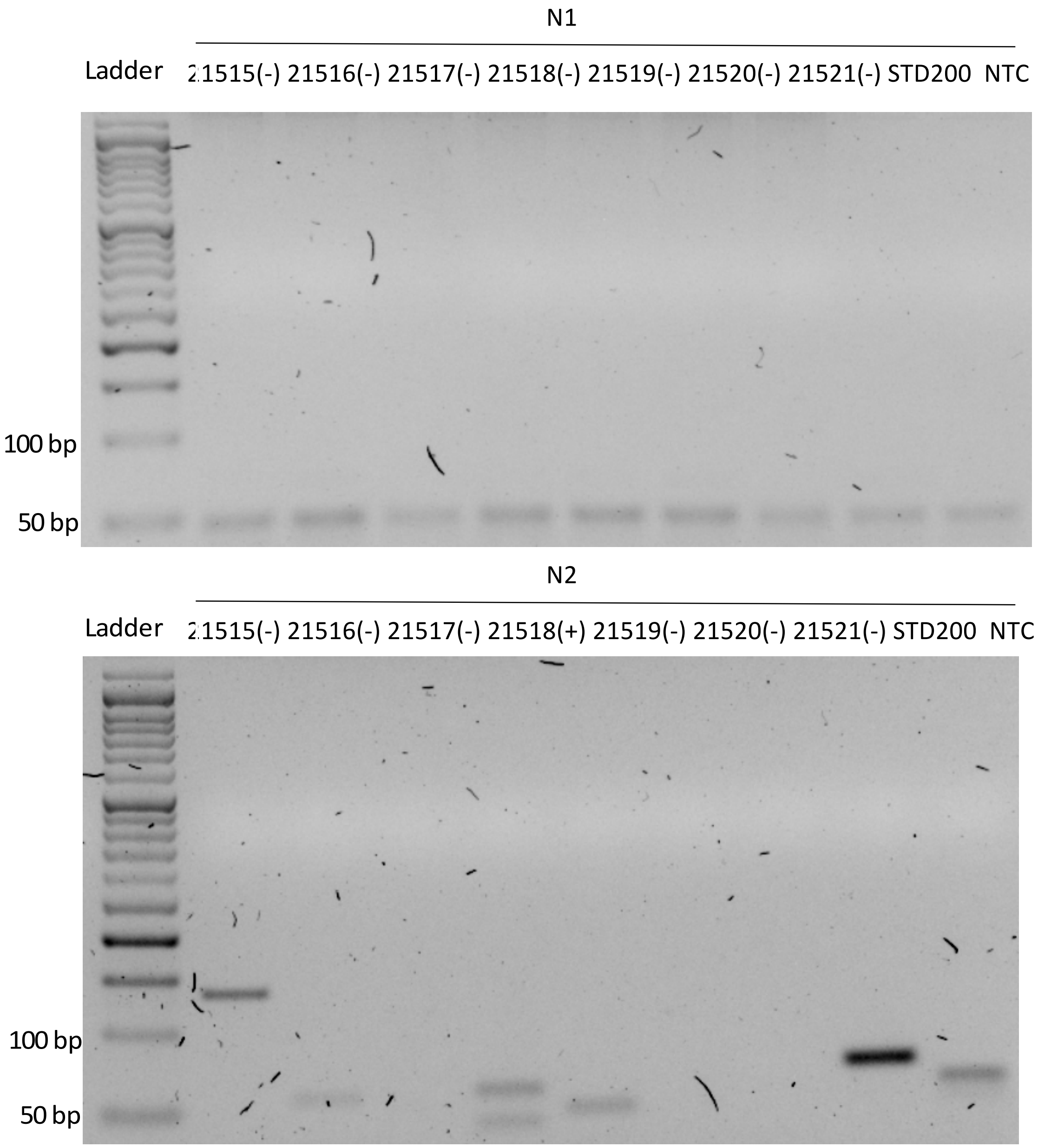
N1
Ladder
21515(-) 21516(-) 21517(-) 21518(-) 21519(-) 21520(-) 21521(-) STD200 NTC
100 bp
50 bp
N2
Ladder
21515(-) 21516(-) 21517(-) 21518(+) 21519(-) 21520(-) 21521(-) STD200 NTC
100 bp
50 bp

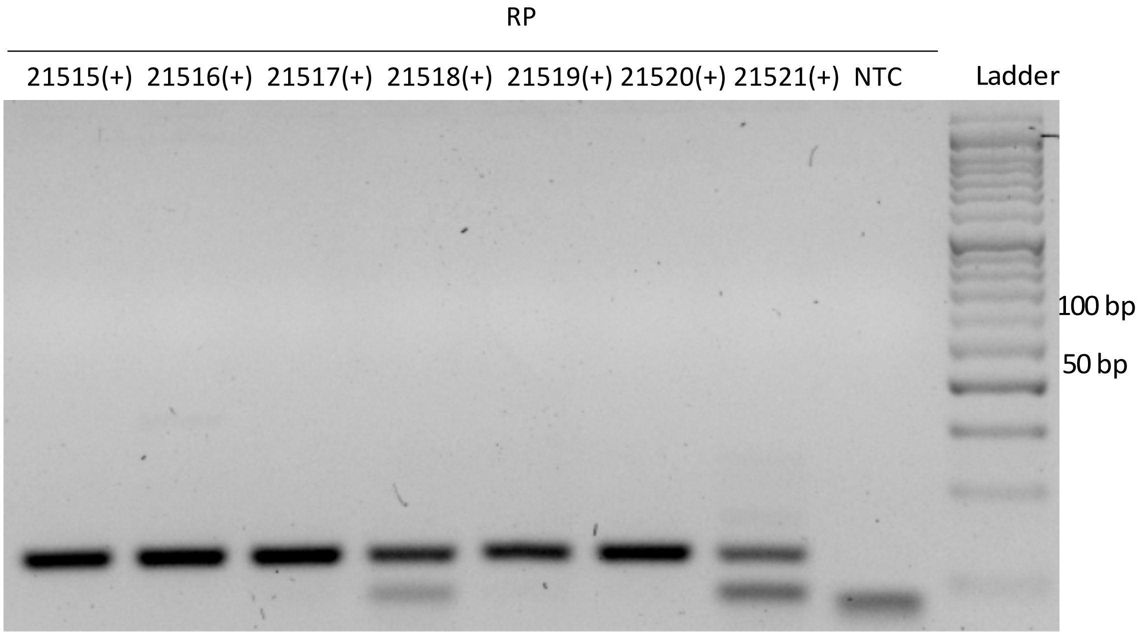
RP
21515(+) 21516(+) 21517(+) 21518(+) 21519(+) 21520(+) 21521(+) NTC
Ladder
100 bp
50 bp

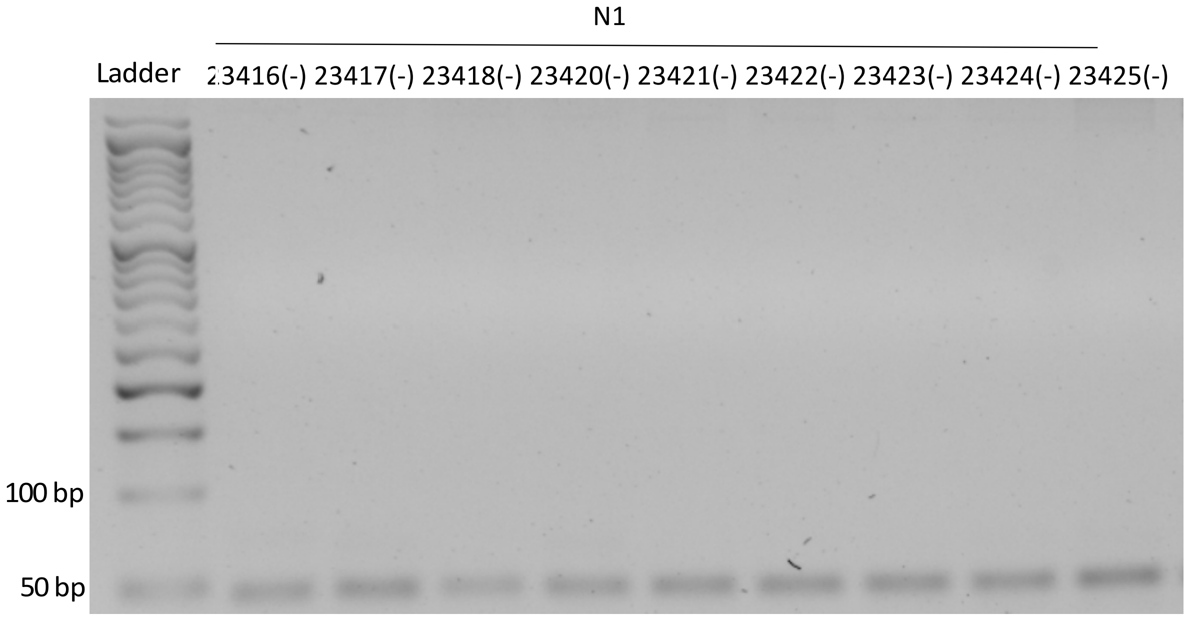
N1
Ladder
23416(-) 23417(-) 23418(-) 23420(-) 23421(-) 23422(-) 23423(-) 23424(-) 23425(-)
100 bp
50 bp

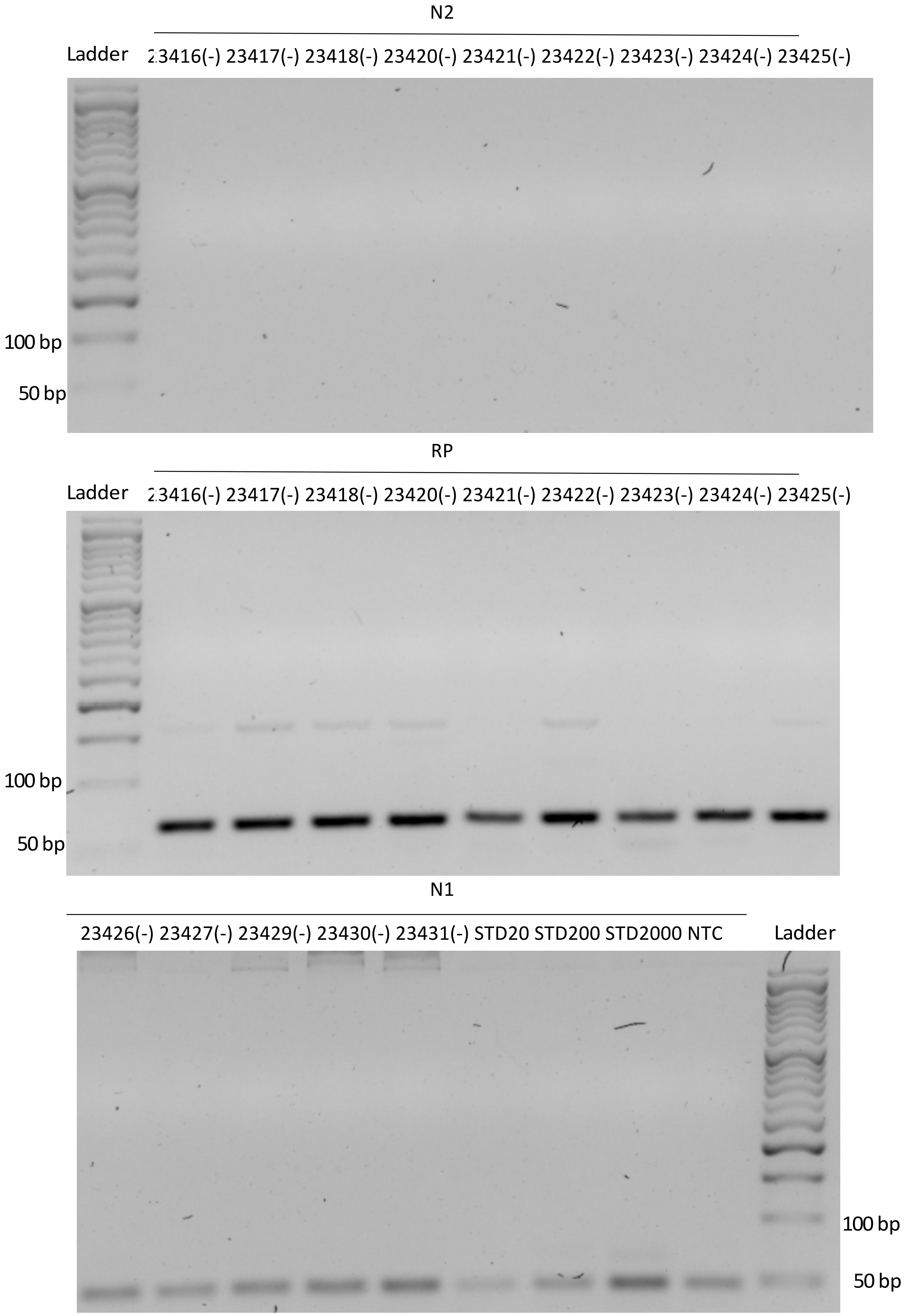
N2
Ladder
23416(-) 23417(-) 23418(-) 23420(-) 23421(-) 23422(-) 23423(-) 23424(-) 23425(-)
100 bp
50 bp
RP
Ladder
23416(-) 23417(-) 23418(-) 23420(-) 23421(-) 23422(-) 23423(-) 23424(-) 23425(-)
100 bp
50 bp
N1
23426(-) 23427(-) 23429(-) 23430(-) 23431(-) STD20 STD200 STD2000 NTC
Ladder
100 bp
50 bp

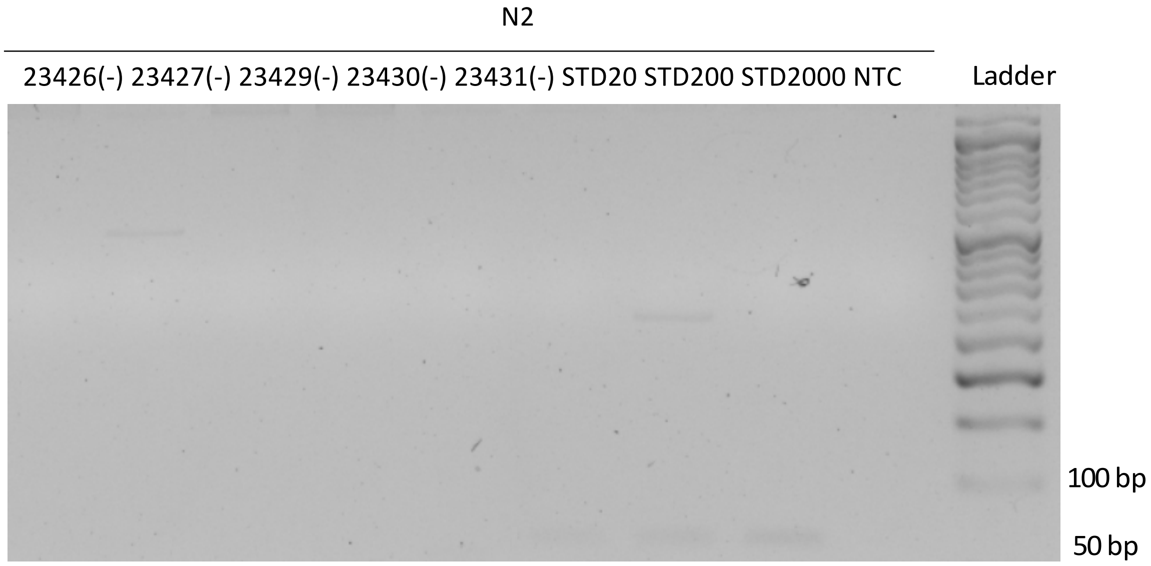
N2
23426(-) 23427(-) 23429(-) 23430(-) 23431(-) STD20 STD200 STD2000 NTC
Ladder
100 bp
50 bp

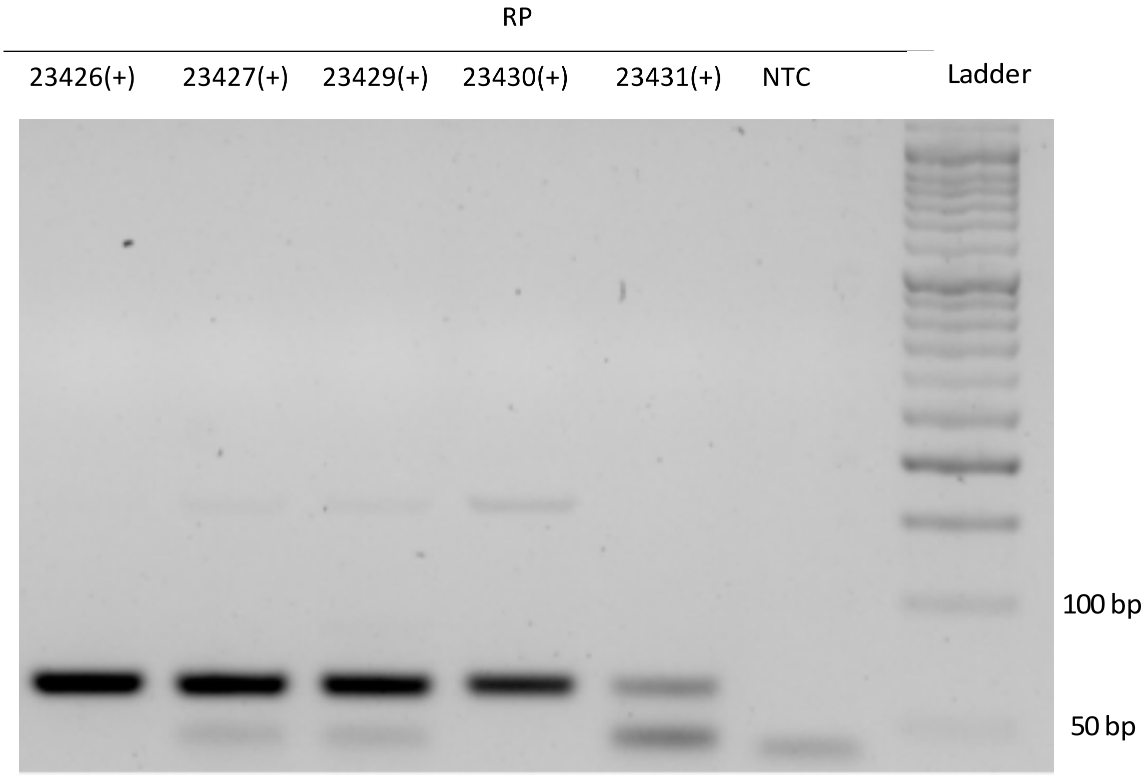
RP
23426(+) 23427(+) 23429(+) 23430(+) 23431(+) NTC
Ladder
100 bp
50 bp

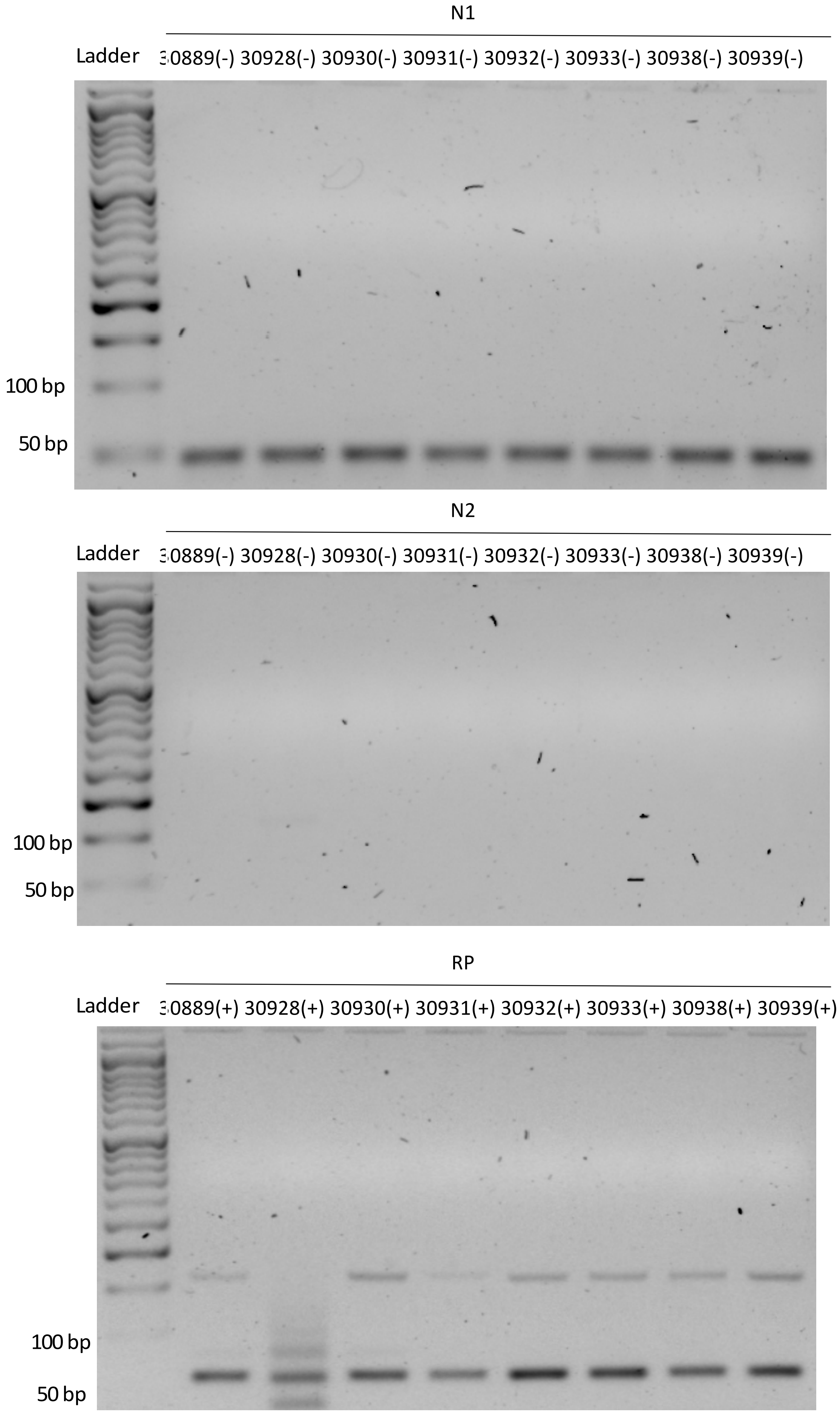
N1
Ladder
0889(-) 30928(-) 30930(-) 30931(-) 30932(-) 30933(-) 30938(-) 30939(-)
100 bp
50 bp
N2
Ladder
0889(-) 30928(-) 30930(-) 30931(-) 30932(-) 30933(-) 30938(-) 30939(-)
100 bp
50 bp
RP
Ladder
0889(+) 30928(+) 30930(+) 30931(+) 30932(+) 30933(+) 30938(+) 30939(+)
100 bp
50 bp

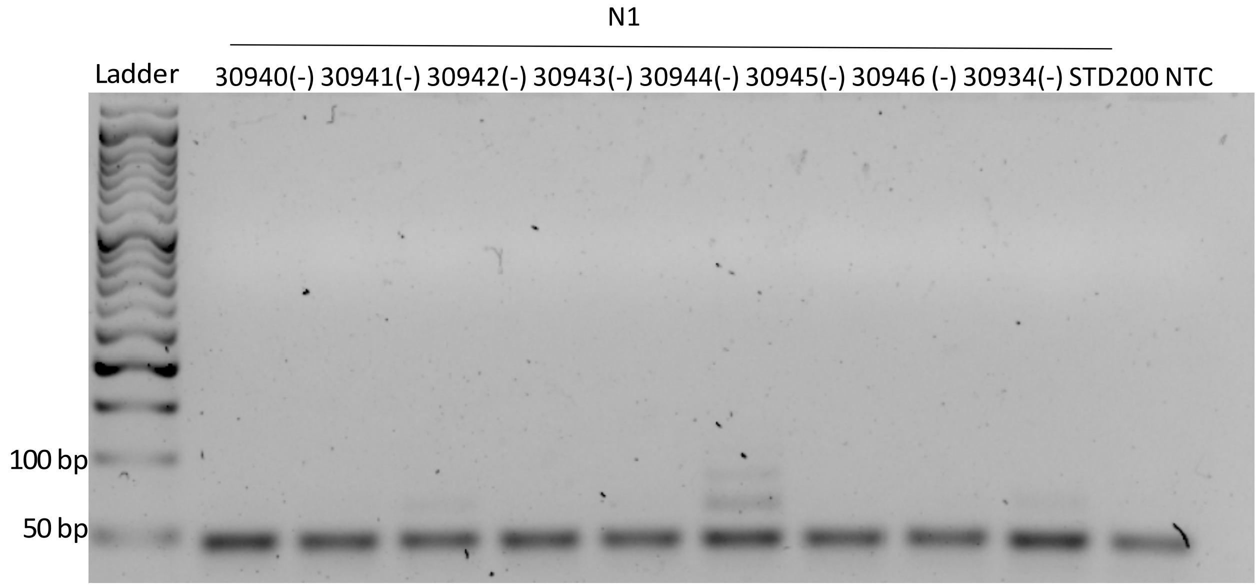
N1
Ladder
30940(-) 30941(-) 30942(-) 30943(-) 30944(-) 30945(-) 30946 (-) 30934(-) STD200 NTC
100 bp
50 bp

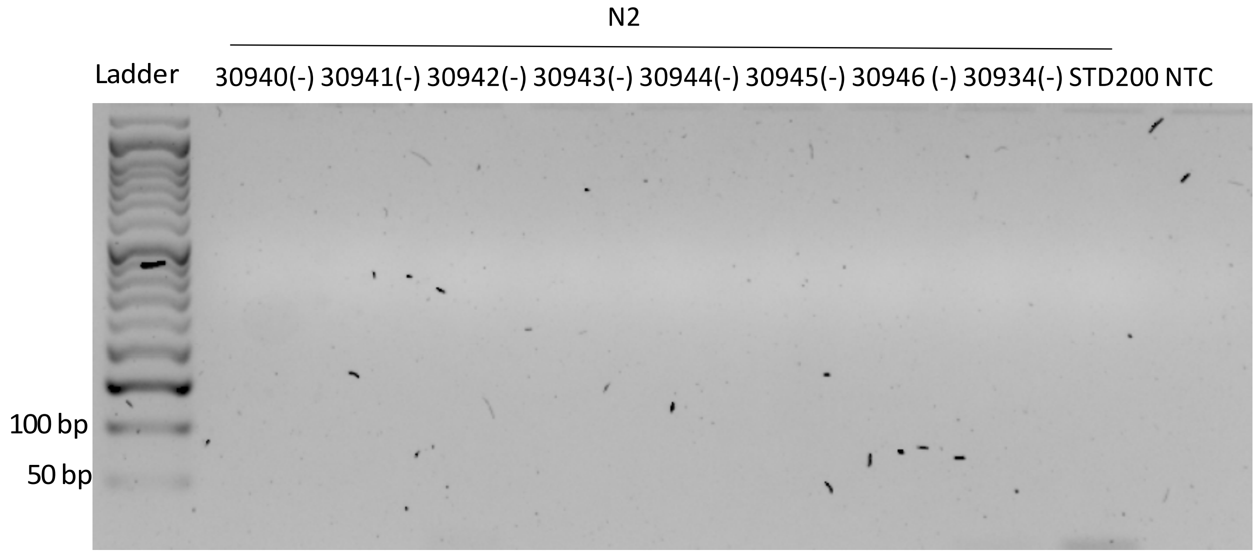
N2
Ladder
30940(-) 30941(-) 30942(-) 30943(-) 30944(-) 30945(-) 30946 (-) 30934(-) STD200 NTC
100 bp
50 bp

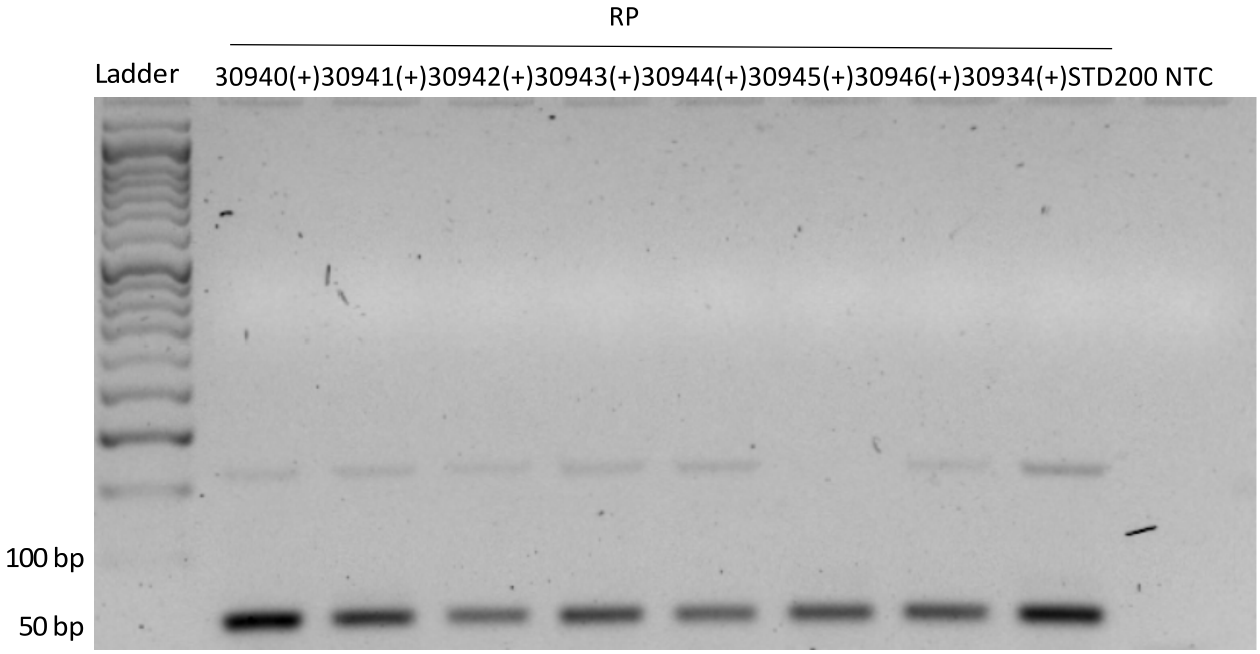
RP
Ladder
30940(+)30941(+)30942(+)30943(+)30944(+)30945(+)30946(+)30934(+)STD200 NTC
100 bp
50 bp

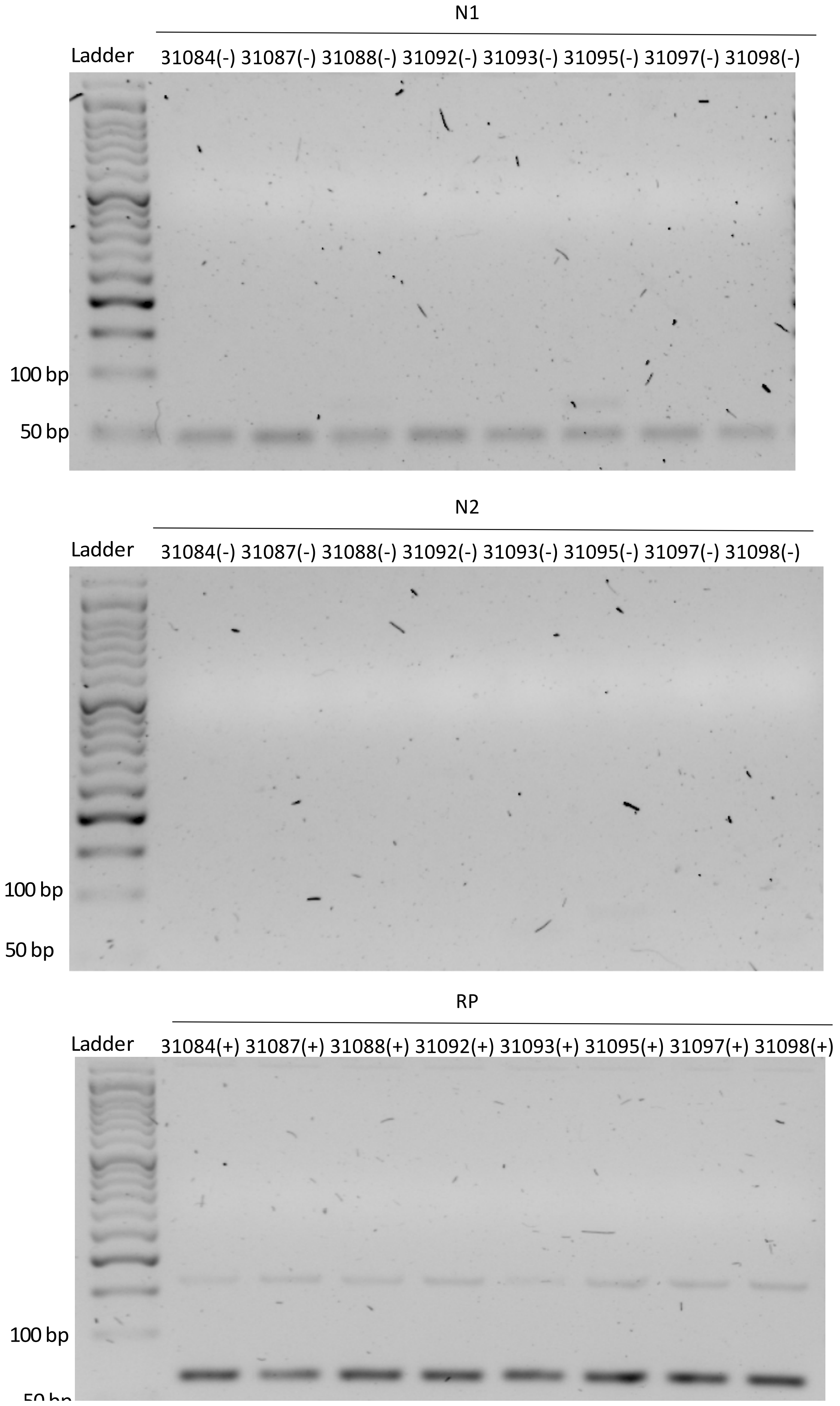
N1
Ladder
31084(-) 31087(-) 31088(-) 31092(-) 31093(-) 31095(-) 31097(-) 31098(-)
100 bp
50 bp
N2
Ladder
31084(-) 31087(-) 31088(-) 31092(-) 31093(-) 31095(-) 31097(-) 31098(-)
100 bp
50 bp
RP
Ladder
31084(+) 31087(+) 31088(+) 31092(+) 31093(+) 31095(+) 31097(+) 31098(+)
100 bp

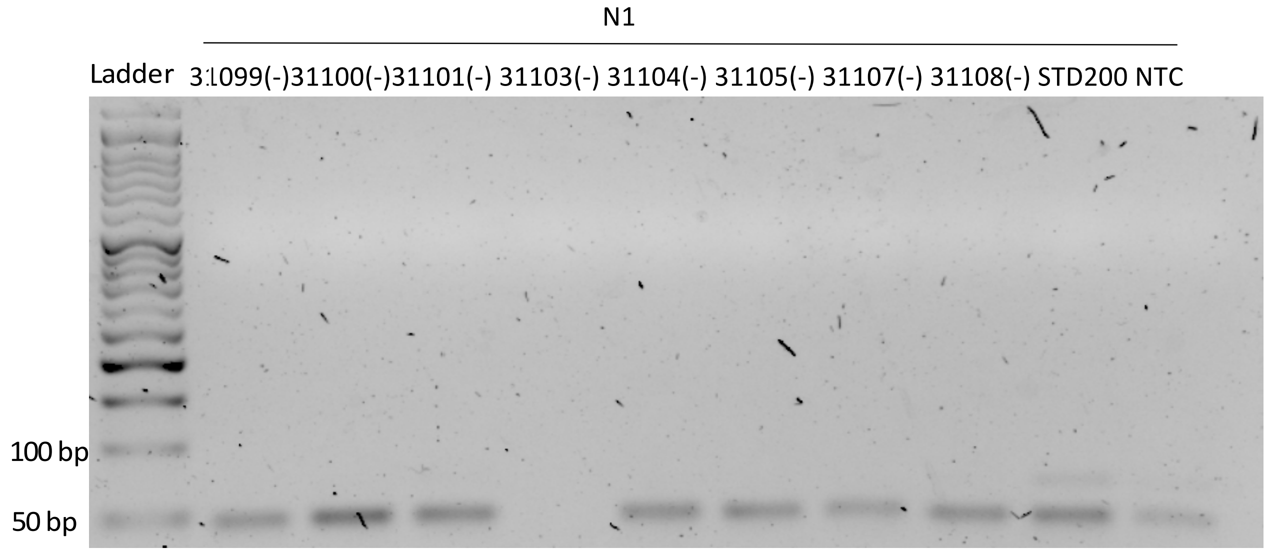
N1
Ladder 31099(-) 31100(-) 31101(-) 31103(-) 31104(-) 31105(-) 31107(-) 31108(-) STD200 NTC
100 bp
50 bp

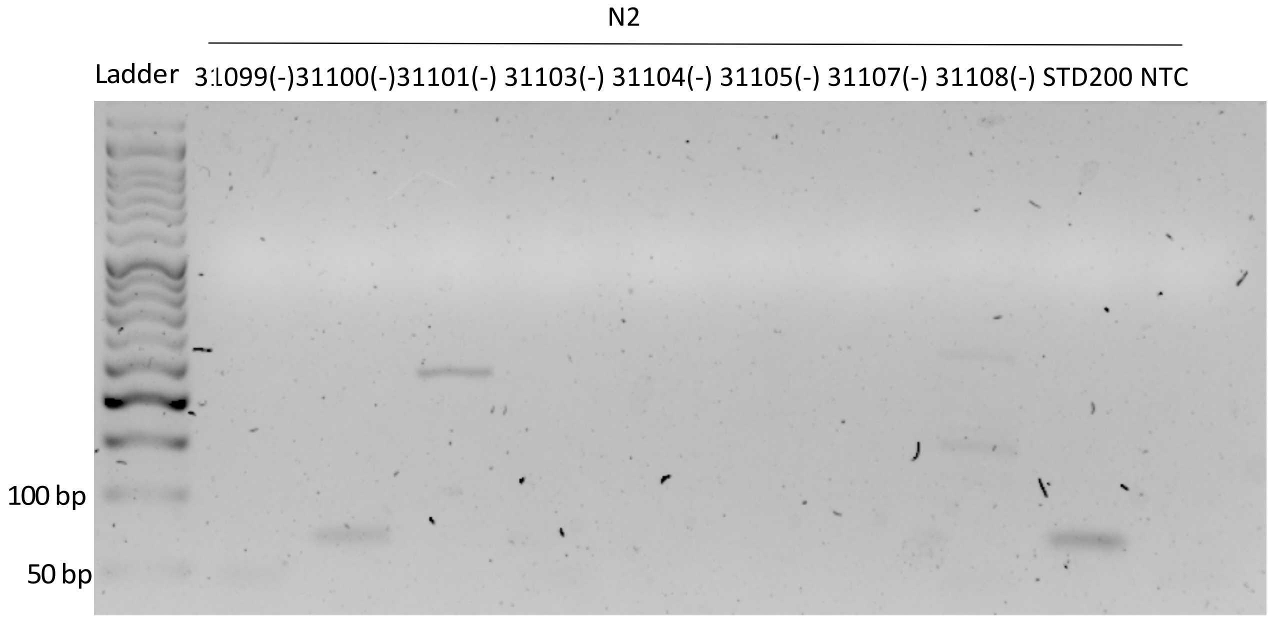
N2
Ladder 31099(-) 31100(-) 31101(-) 31103(-) 31104(-) 31105(-) 31107(-) 31108(-) STD200 NTC
100 bp
50 bp

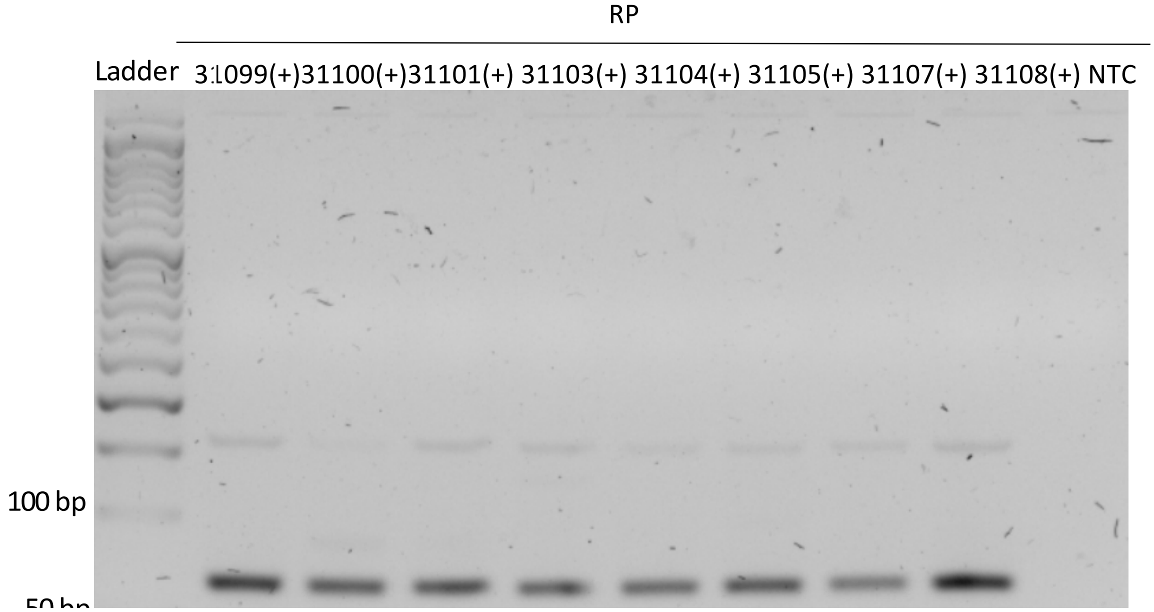
RP
Ladder 31099(+) 31100(+) 31101(+) 31103(+) 31104(+) 31105(+) 31107(+) 31108(+) NTC
100 bp

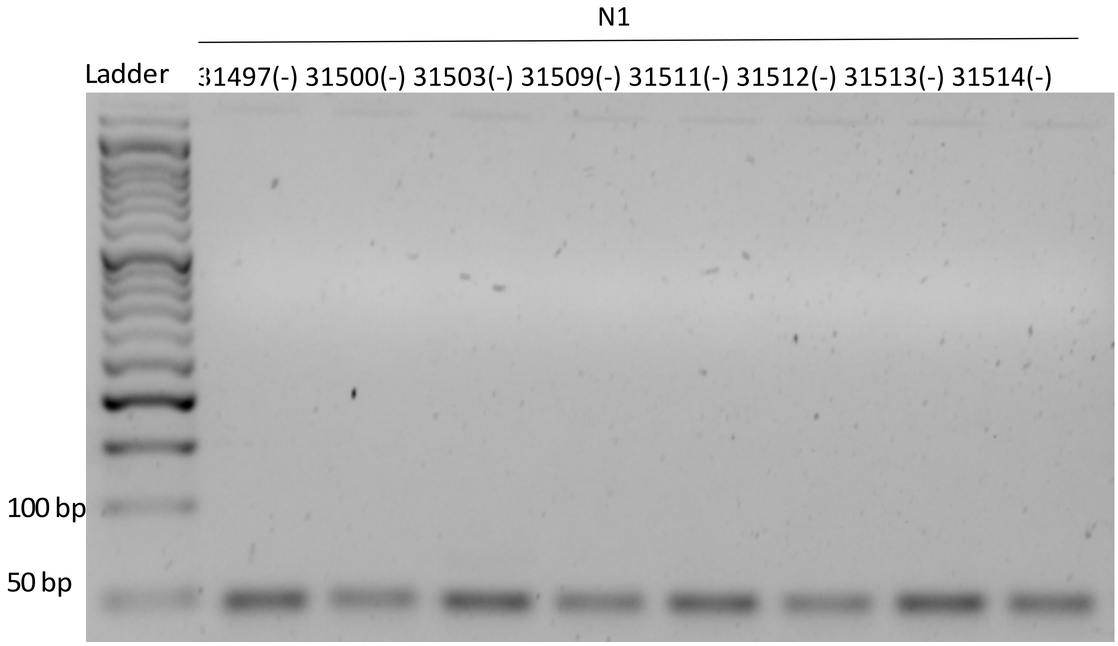
N1
Ladder
31497(-) 31500(-) 31503(-) 31509(-) 31511(-) 31512(-) 31513(-) 31514(-)
100 bp
50 bp

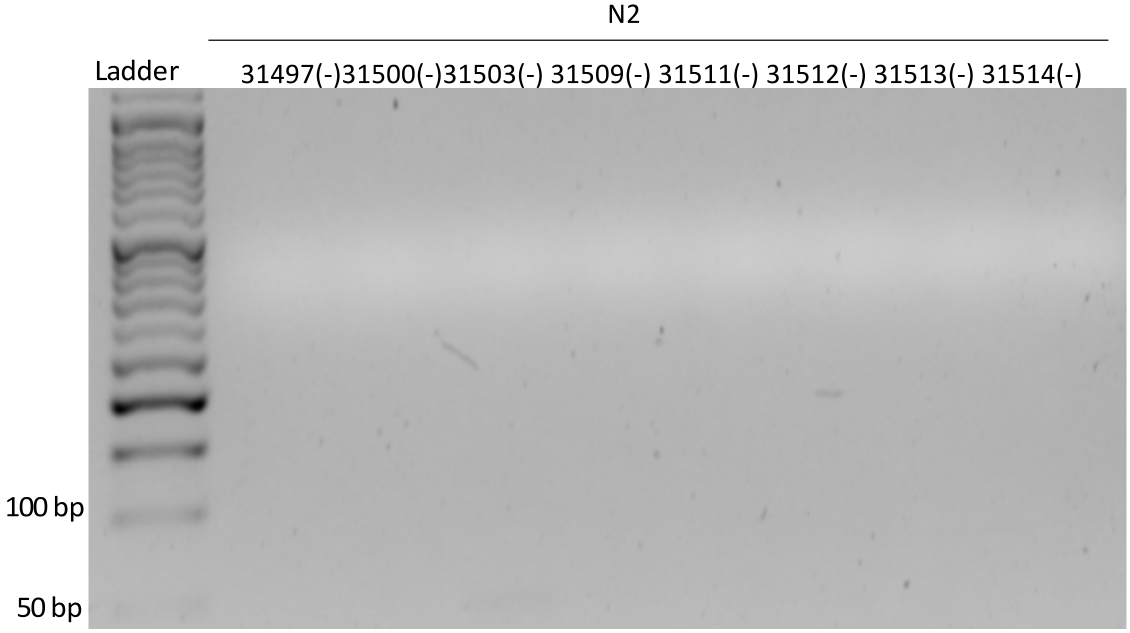
N2
Ladder
31497(-)31500(-)31503(-) 31509(-) 31511(-) 31512(-) 31513(-) 31514(-)
100 bp
50 bp

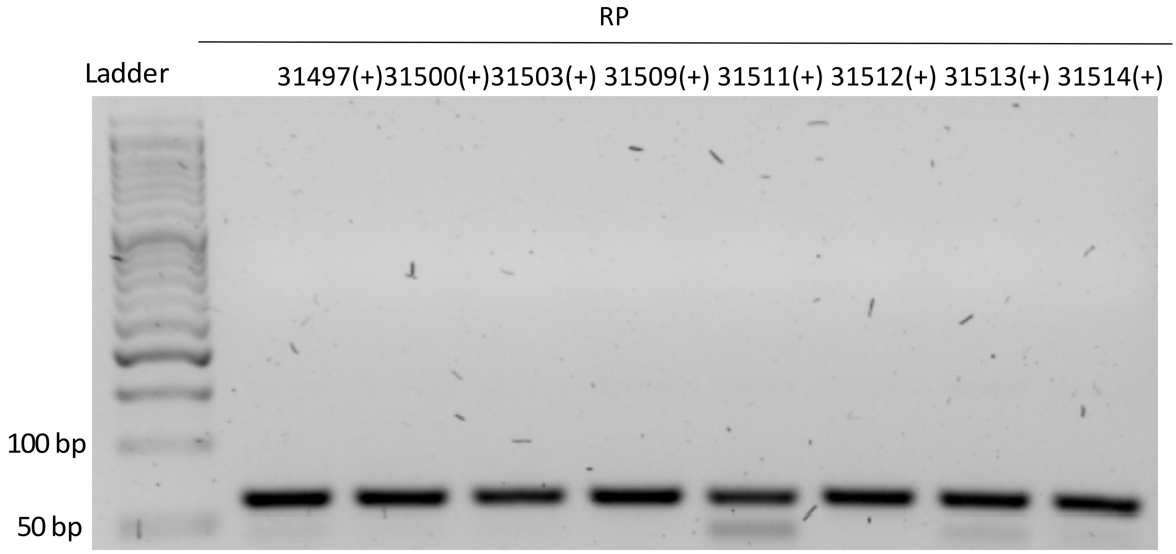
RP
Ladder
31497(+)31500(+)31503(+) 31509(+) 31511(+) 31512(+) 31513(+) 31514(+)
100 bp
50 bp

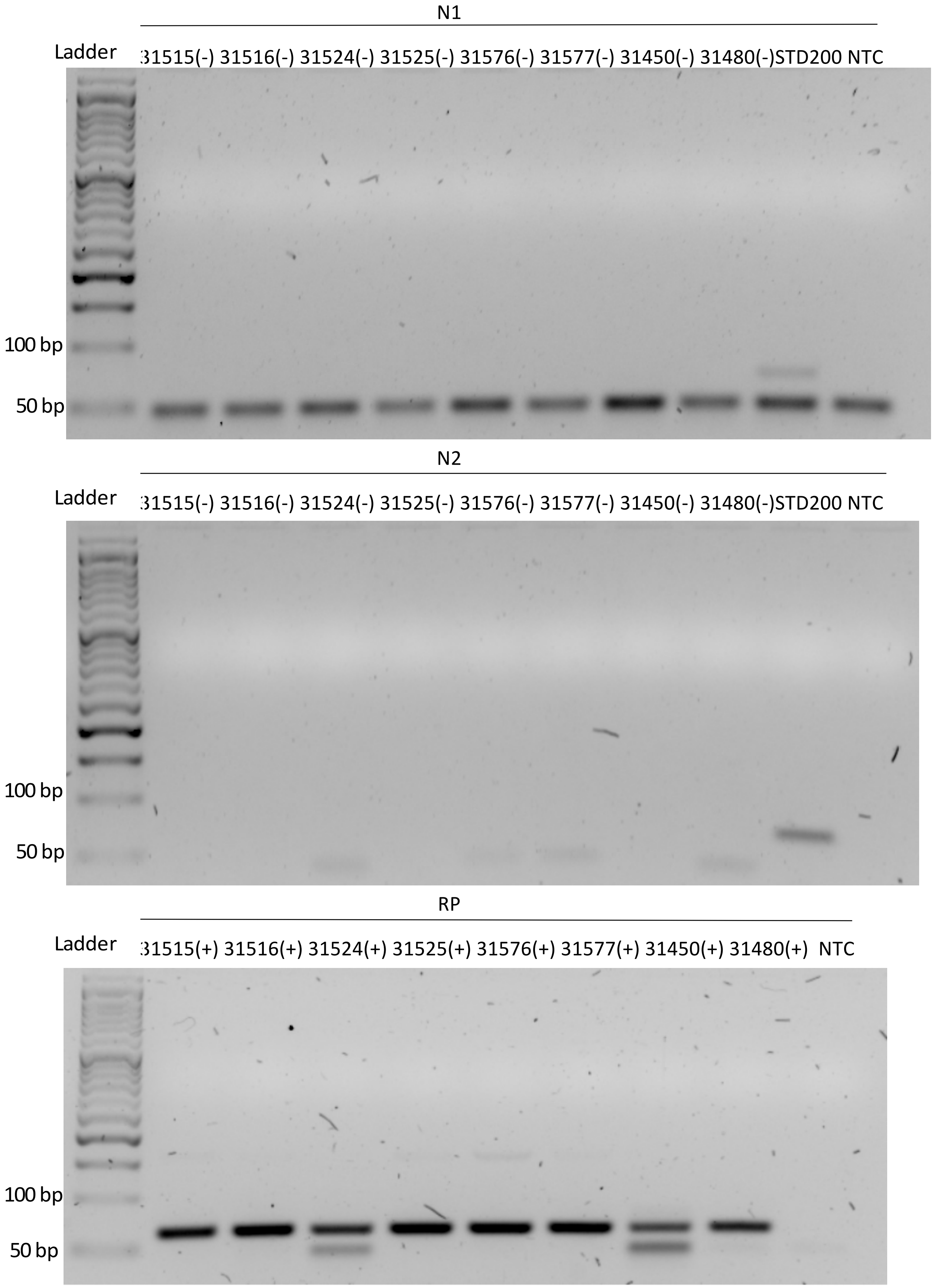

N1
Ladder
31515(-) 31516(-) 31524(-) 31525(-) 31576(-) 31577(-) 31450(-) 31480(-) STD200 NTC
100 bp
50 bp
N2
Ladder
31515(-) 31516(-) 31524(-) 31525(-) 31576(-) 31577(-) 31450(-) 31480(-) STD200 NTC
100 bp
50 bp
RP
Ladder
31515(+) 31516(+) 31524(+) 31525(+) 31576(+) 31577(+) 31450(+) 31480(+) NTC
100 bp
50 bp

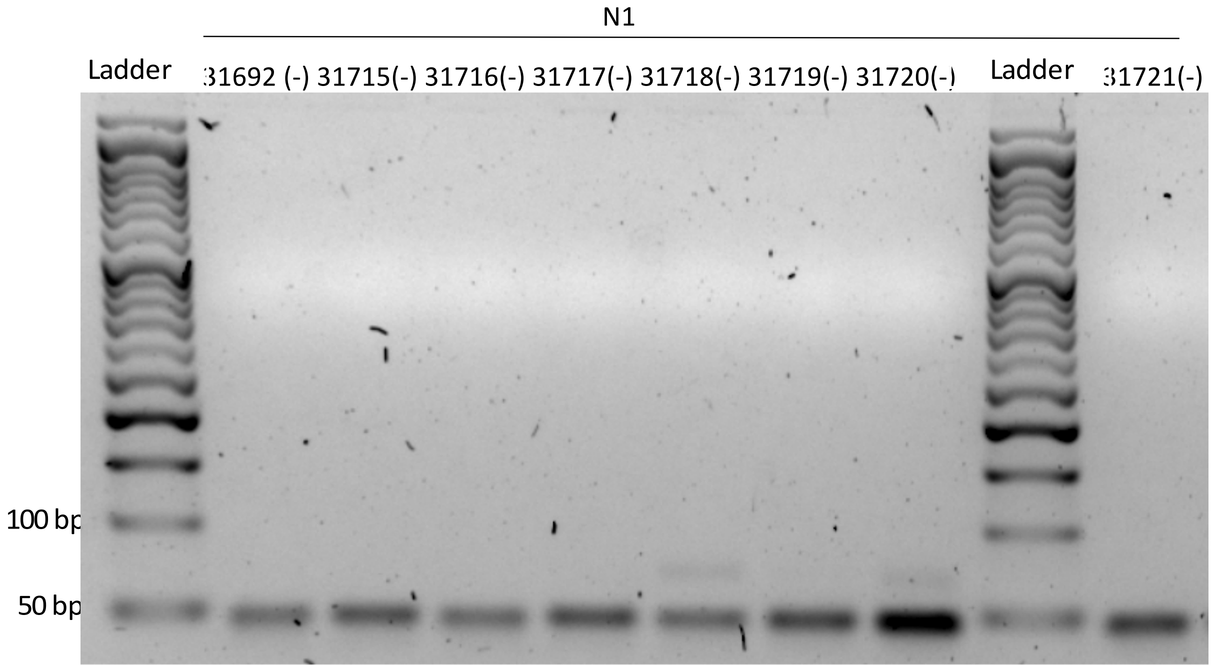
N1
Ladder
31692 (-)
31715(-)
31716(-)
31717(-)
31718(-)
31719(-)
31720(-)
Ladder
31721(-)
100 bp
50 bp

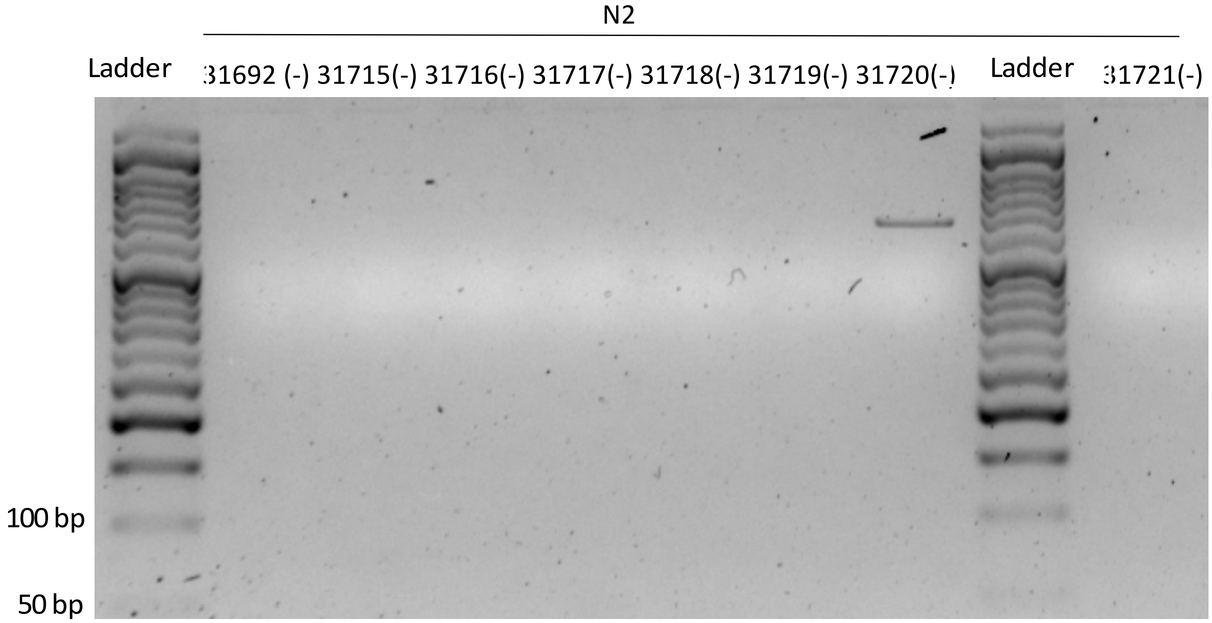
N2
Ladder
31692 (-)
31715(-)
31716(-)
31717(-)
31718(-)
31719(-)
31720(-)
Ladder
31721(-)
100 bp
50 bp

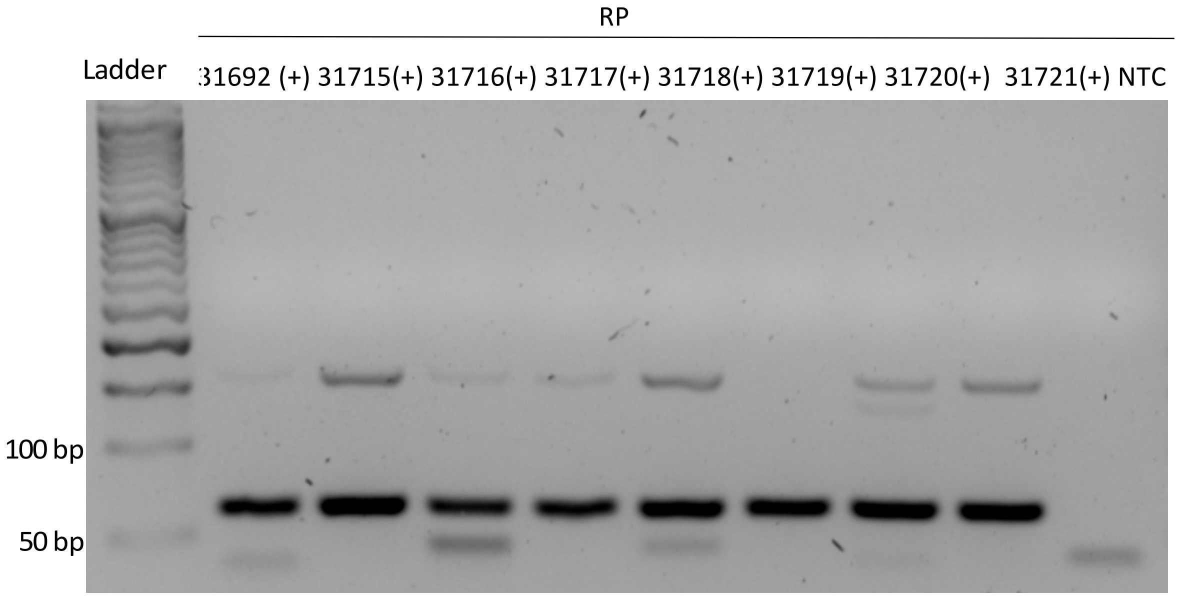
RP
Ladder
31692 (+)
31715(+)
31716(+)
31717(+)
31718(+)
31719(+)
31720(+)
31721(+)
NTC
100 bp
50 bp
