# Supplementary material for: High sensitivity-low cost detection of SARS-CoV-2 by two steps end point RT-PCR with agarose gel electrophoresis visualization
Source: Sci Rep. 2021 Nov 4;11:21658. doi: 10.1038/s41598-021-00900-8 (PMC8568942; doi:10.1038/s41598-021-00900-8)

Supplementary material 3. Full agarose gel electrophoresis pictures for samples included on the study that were used to make Figure 1. Only in this set of samples, viral target N3 was also included. The samples codes for all this samples are detailed on Supplementary Material 1, where there is a column indicating if samples were used for Figure 1.

12 SARS-CoV-2 positive samples for N1, N2, N3 and RNaseP end point PCR (Samples 1-12 on Suplementary material 1)

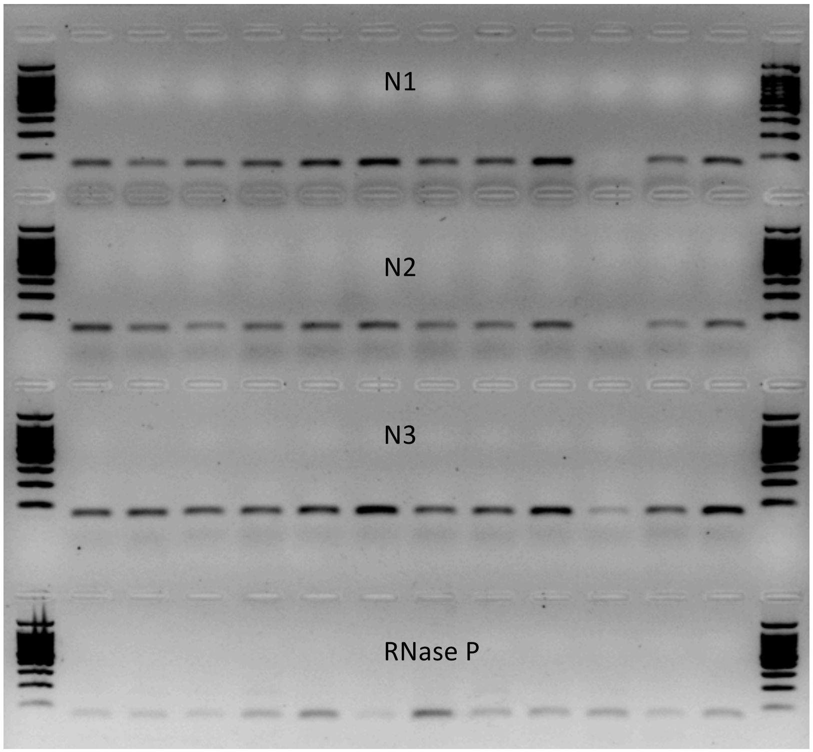

12 SARS-CoV-2 negative samples for N1, N2. N3 and RNaseP end point PCR (Samples 123 to 135 on Supplementary Material 1)

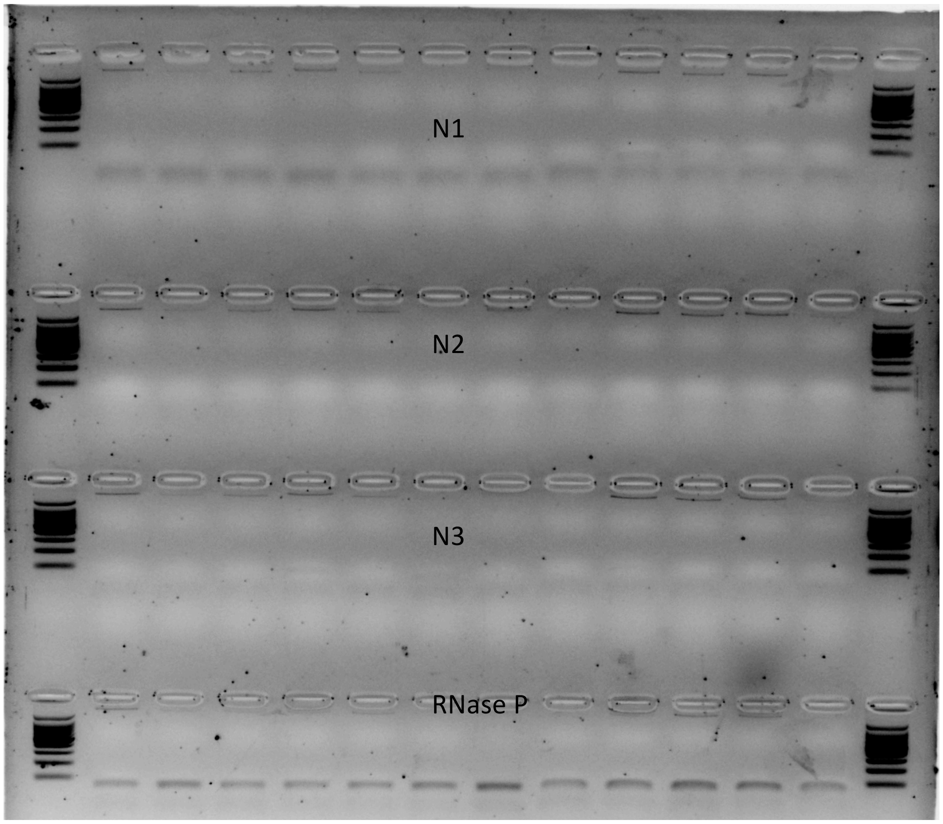

Supplement: Supplementary file 3 — Supplementary Information 3. [file 41598_2021_900_MOESM3_ESM.pdf]
