# Supplementary material for: High sensitivity-low cost detection of SARS-CoV-2 by two steps end point RT-PCR with agarose gel electrophoresis visualization
Source: Sci Rep. 2021 Nov 4;11:21658. doi: 10.1038/s41598-021-00900-8 (PMC8568942; doi:10.1038/s41598-021-00900-8)

Supplementary Material 4. Agarose gels for other respiratory viruses to assess cross reactivity with N1 and N2 viral targets.

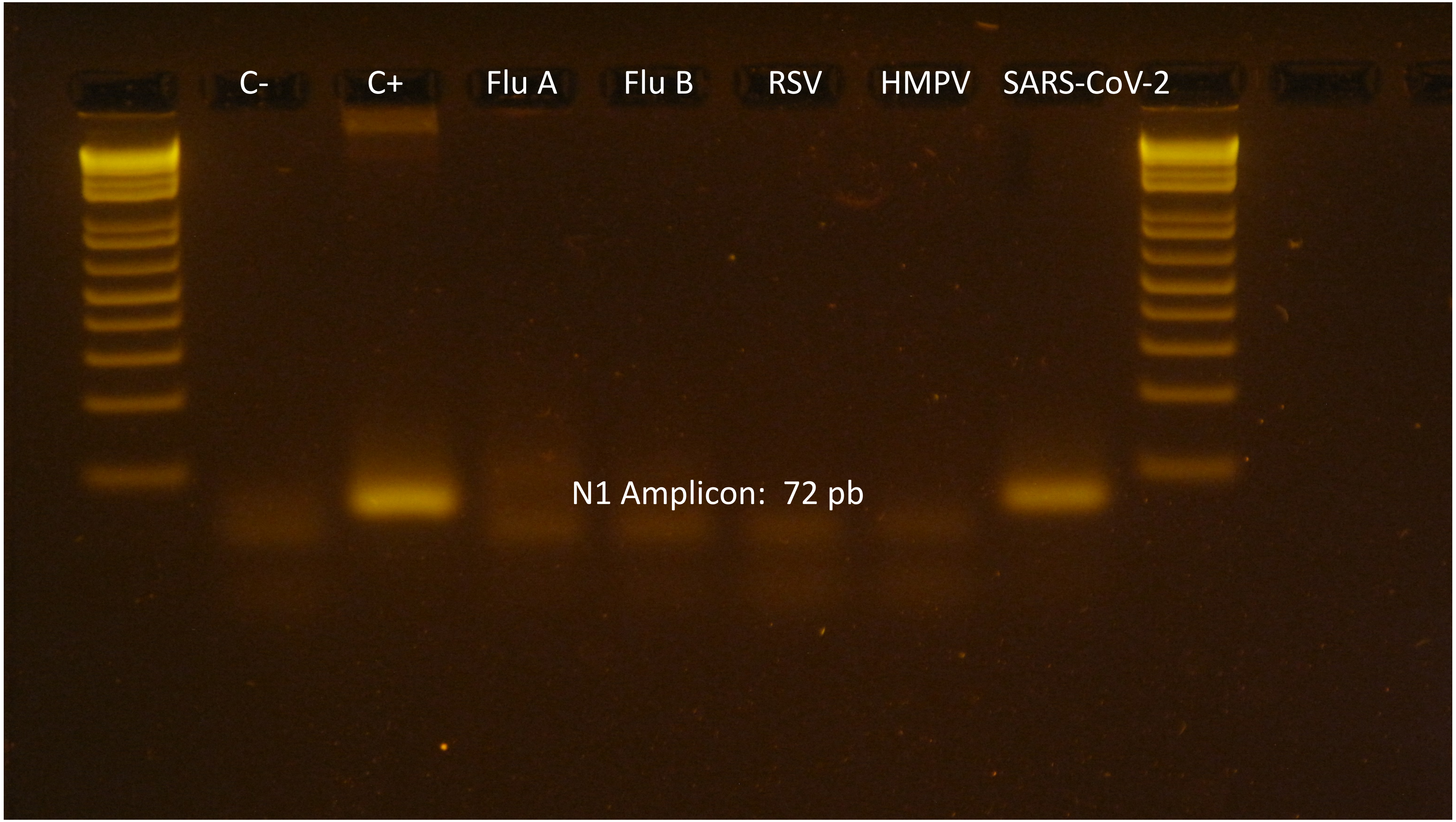

Supplementary Material 4. Agarose gels for other respiratory viruses to assess cross reactivity with N1 and N2 viral target.

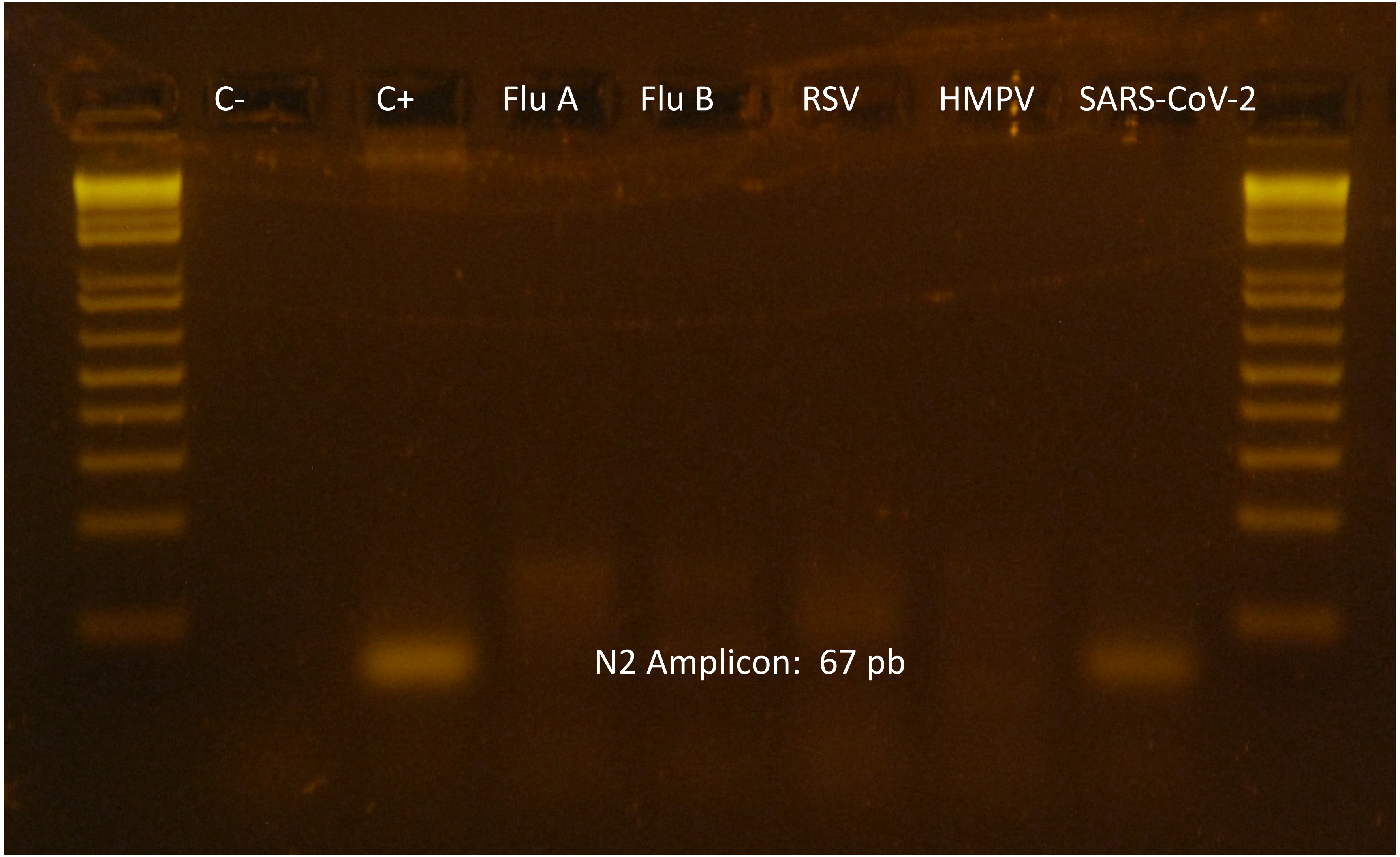

Supplement: Supplementary file 4 — Supplementary Information 4. [file 41598_2021_900_MOESM4_ESM.pdf]
